# Nuclear Export and Import of Human Hepatitis B Virus Capsid Protein and Particles

**DOI:** 10.1371/journal.ppat.1001162

**Published:** 2010-10-28

**Authors:** Hung-Cheng Li, Er-Yi Huang, Pei-Yi Su, Szu-Yao Wu, Ching-Chun Yang, Young-Sun Lin, Wen-Chang Chang, Chiaho Shih

**Affiliations:** 1 Institute of Biochemical Sciences, National Taiwan University, Taipei, Taiwan; 2 Institute of Biomedical Sciences, Academia Sinica, Taipei, Taiwan; 3 Graduate Institute of Molecular Medicine, School of Medicine, National Taiwan University, Taipei, Taiwan; 4 Graduate Institute of Microbiology and Immunology, National Yang-Ming University, Taipei, Taiwan; University of Southern California, United States of America

## Abstract

It remains unclear what determines the subcellular localization of hepatitis B virus (HBV) core protein (HBc) and particles. To address this fundamental issue, we have identified four distinct HBc localization signals in the arginine rich domain (ARD) of HBc, using immunofluorescence confocal microscopy and fractionation/Western blot analysis. ARD consists of four tight clustering arginine-rich subdomains. ARD-I and ARD-III are associated with two co-dependent nuclear localization signals (NLS), while ARD-II and ARD-IV behave like two independent nuclear export signals (NES). This conclusion is based on five independent lines of experimental evidence: **i**) Using an HBV replication system in hepatoma cells, we demonstrated in a double-blind manner that only the HBc of mutant ARD-II+IV, among a total of 15 ARD mutants, can predominantly localize to the nucleus. **ii**) These results were confirmed using a chimera reporter system by placing mutant or wild type HBc trafficking signals in the heterologous context of SV40 large T antigen (LT). **iii**) By a heterokaryon or homokaryon analysis, the fusion protein of SV40 LT-HBc ARD appeared to transport from nuclei of transfected donor cells to nuclei of recipient cells, suggesting the existence of an NES in HBc ARD. This putative NES is leptomycin B resistant. **iv**) We demonstrated by co-immunoprecipitation that HBc ARD can physically interact with a cellular factor TAP/NXF1 (Tip-associated protein/nuclear export factor-1), which is known to be important for nuclear export of mRNA and proteins. Treatment with a TAP-specific siRNA strikingly shifted cytoplasmic HBc to nucleus, and led to a near 7-fold reduction of viral replication, and a near 10-fold reduction in HBsAg secretion. **v**) HBc of mutant ARD-II+IV was accumulated predominantly in the nucleus in a mouse model by hydrodynamic delivery. In addition to the revised map of NLS, our results suggest that HBc could shuttle rapidly between nucleus and cytoplasm via a novel TAP-dependent NES.

## Author Summary

Chronic infection with hepatitis B virus (HBV) could lead to cirrhosis and highly malignant liver cancer. At present, treatment of hepatitis B is not very effective, due to notorious side effects and drug resistance. The virus can synthesize a core protein for its own replication. Clinically, this core protein tends to be more localized to the cytoplasm when patients' disease is severe, and more localized to the nucleus when the disease is mild. To date, the rules that govern the partition of the intracellular core protein into nucleus vs. cytoplasm remain unclear. Using 5 independent approaches, we identified the “entry passport” (nuclear localization signal) for the core protein to enter the nucleus. Surprisingly, we also encountered an unexpected “exit passport” (nuclear export signal) for the core protein to exit the nucleus. A host factor coined TAP can serve as a shuttling bus, which can take passengers like the core protein by its ticket (exit passport), and assist it to leave the nucleus. We proposed that this core protein does not “park” in the cytoplasm. Rather, it is shuttling continuously between nucleus and cytoplasm, in order to amplify viral DNA. When we reduced the TAP protein, HBV replication was dramatically reduced. Our finding could facilitate drug discovery against hepatitis B.

## Introduction

Hepatitis B virus (HBV) is an important human pathogen [1], [2], [3]. Despite the success of HBV vaccination program in certain countries [4], there is no nationwide vaccination program in most endemic areas in Asia and Africa. Vaccine failure and non-responsive cases are not uncommon [5]. Longer term treatment of HBV patients with nucleoside/nucleotide analogs often results in drug resistance [6]. Interferon is expensive and has notorious side effects. Chronic infection with HBV remains to be a major etiological factor for cirrhosis, liver failure, and hepatocellular carcinoma [1], [2], [3].

HBV encodes a 21 kDa core (HBc or capsid) protein, which is required for reverse transcription [7], [8]. This core protein can assemble into stable icosahedral particles, when the electrostatic interactions between positively and negatively charged macromolecules at the inner surface of the capsid shell are adequately balanced [9], [10], [11]. The intracellular core particles can partition into nucleus and cytoplasm [12], [13]. Clinically, hepatic HBc in patients with minor hepatitis activity was distributed mainly in the nuclei. In contrast, in patients with chronic active liver diseases, hepatic HBc was distributed predominantly in the cytoplasm [14], [15], [16], [17], [18]. Mature cytoplasmic core particles contain relaxed circle (RC) DNA genome, which needs to be transported to nucleus for conversion into covalently closed circular DNA [7], [8].

**Figure 1:**
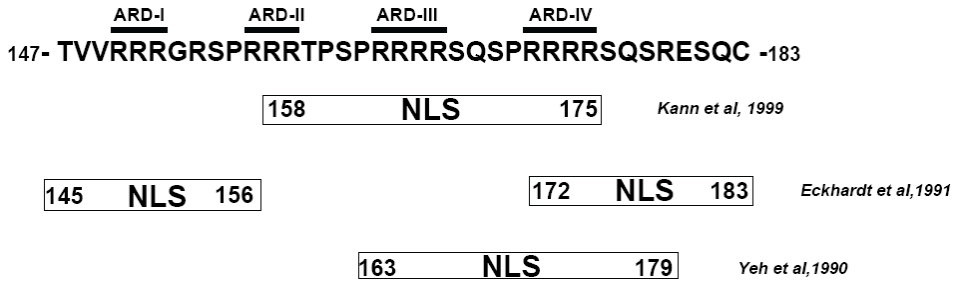
The mapped locations of HBc nuclear localization signals reported in literature are contradictory [23], [24], [25].

The molecular mechanism of HBc nuclear import has remained unclear. The arginine rich domain (ARD) at the C-terminus of HBc 147–183 contains four stretches of clustering arginines (Fig. 1). The amino acid sequences of these clustering arginines resemble the structural features of known SR proteins [19] or nuclear localization signals (NLS) [20], [21], [22]. As shown in Fig. 1, Yeh et al. [23] reported that at least two direct repeats of PRRRRSQS (spanning ARD-III and ARD-IV) are required for nuclear import of HBc. One copy alone of the sequence PRRRRSQS is not sufficient for HBc nuclear import. On the other hand, it has also been reported that ARD-I and ARD-IV are associated with two independent NLS of HBc in stably transfected mouse NIH3T3 fibroblasts. ARD-I alone, or ARD-IV alone, is sufficient for HBc nuclear localization [24]. Kann et al. [25] demonstrated that HBc particles can bind to nuclear pore complex (NPC) in digitonin-permeabilized mammalian cells. NPC binding of HBc capsids was inhibited by synthetic peptides HBc 158–168 or 165–175, suggesting that part of ARD-II, ARD-III, and/or ARD-IV, may contain the potential NLS. To date, the discrepancy of the exact location of HBc NLS remains to be resolved. Furthermore, previous studies on this issue were performed in the context of HBc expression without HBV viral replication [23], [24], [25]. It is known that more predominant cytoplasmic HBc tends to be associated with more active HBV DNA replication [14], [15], [16], [17], [18].

Here, we took a new approach to revisit an old issue of HBc trafficking by using a Huh7 hepatoma cell line permissive for active HBV replication. We mapped two co-dependent NLS-I and NLS-III to new positions containing ARD-I and ARD-III, respectively. Most interestingly, we disclosed two unexpected nuclear export signals (NES-II and NES-IV)/cytoplasmic retention signals (CRS-II and CRS-IV), each of which could function independently from the other. The same HBc ARD could function as a CRS or NES, depending on the relative strength between NLS and NES in the context. Furthermore, we demonstrated here physical and functional interactions between a cellular factor TAP/NXF1 and HBc ARD. TAP (Tip associated protein) was identified by a yeast two-hybrid screen using Tip of herpesvirus saimiri as bait [26]. As a nucleocytoplasmic shuttling protein, TAP is also known as a major receptor for bulk mRNP export [27], [28]. Our results support the notion that HBc could be shuttling between nucleus and cytoplasm rapidly and continuously. Furthermore, the significant reduction of HBV DNA synthesis by siTAP treatment could offer a new avenue for HBV therapy in the future. Finally, we discussed about a potential role of HBc nucleocytoplasmic shuttling in HBV pathobiology, such as acute exacerbation or spontaneous relapse in HBV chronic carriers.

## Materials and Methods

### Construction of fifteen arginine-to alanine (R-to-A) HBc ARD mutants

**Figure 2:**
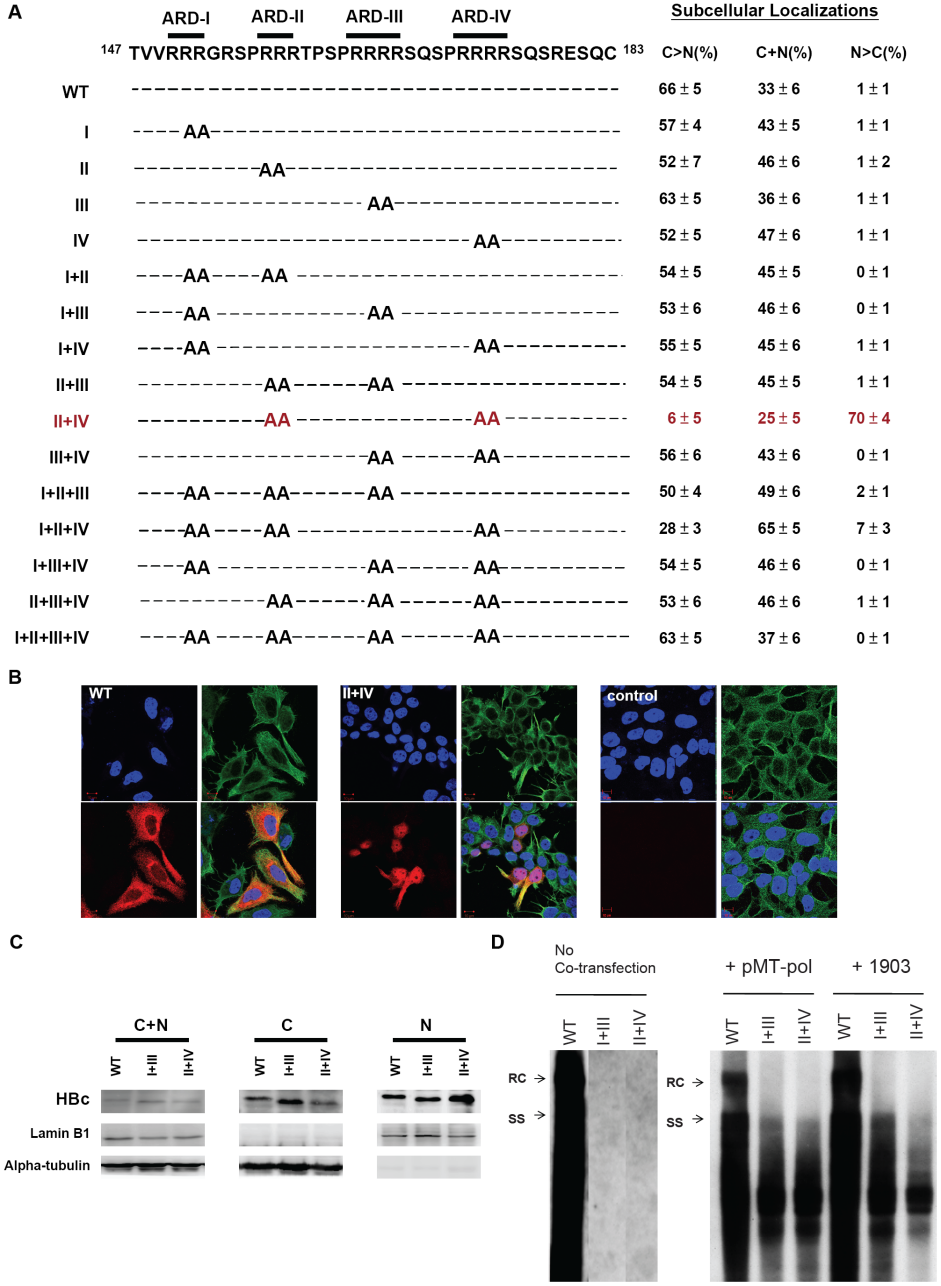
Subcellular localization of HBc of 15 HBc ARD mutants was evaluated by immunofluorescence assay (IFA) of Huh7 cells, which had been cotransfected with HBc ARD mutants and plasmid pMT-pol expressing HBV polymerase, using a double-blind protocol. **Fig. 2A**. Amino acid sequences of 15 alanine scanning ARD mutants are shown in the left panel. In the right panel, the pattern “C>N” represents cells which exhibited predominant cytoplasmic HBc, with weak or no detectable nuclear HBc. In contrast, the pattern “N>C” represents cells which exhibited predominant nuclear HBc, with weak or no detectable cytoplasmic HBc. The pattern “C+N” represents a third case when HBc was present in both cytoplasm and nucleus, with no strong preference for either compartment. A summary of the IFA results of these ARD mutants revealed that only mutant HBc ARD-II+IV exhibited an apparent phenotype in HBc nuclear targeting (mutant HBc ARD-I+II+IV exhibited a very weak nuclear targeting phenotype). The average numbers and standard deviations are taken from the results of three independent double-blind experiments. **Fig. 2B**. Immunofluorescence assay of Huh7 cells was performed using confocal microscopy. HBc (red), α-tubulin (green), and DAPI (blue). **Fig. 2C**. Increased nuclear accumulation of HBc of mutant ARD-II+IV was confirmed by fractionation and Western blot analysis using anti-HBc antibody. The altered electrophoretic mobility on SDS-PAGE of mutant HBc ARD-I+III, and ARD-II+IV, is likely due to their loss of four arginine residues, which resulted in an altered charge/mass ratio of the mutant HBc proteins. **Fig. 2D**. Southern blot analysis of HBV DNA replication of wild type HBV, mutant ARD-I+III, and mutant ARD-II+IV in Huh7 cells. Both mutant ARD-I+III and mutant ARD-II+IV are completely replication defective by themselves. However, the replication defect of mutant ARD-I+III and mutant ARD-II+IV can be partially rescued by cotransfection with plasmid pMT-pol, or plasmid 1903. In the left panel (no cotransfection), all three lanes were derived from the same gel. The first lane of wild type HBV DNA was overexposed on purpose, in order to visualize any weak bandings in lanes I+III and II+IV, if any. See text for further discussion.

A total of 15 HBc ARD mutants were constructed by QuickChange XL Site-Directed Mutagenesis (Stratagene Co.), using the wild type HBV DNA (subtype *ayw*) of plasmid pCH93091 as a template. This plasmid cannot produce precore and HBeAg, but is replication competent [29]. Arginine-to-alanine substitutions were introduced into HBc amino acid 151 and 152 of mutant ARD-I, 157 and 158 of mutant ARD-II, 165 and 166 of mutant ARD-III, and 173 and 174 of mutant ARD-IV. All mutants were confirmed by DNA sequencing. Most of these 15 ARD mutants are replication defective by Southern blot analysis (Fig. 2D; manuscript in preparation), and can be rescued to replicate to various extents by cotransfection with plasmid pMT-pol [30], or with plasmid 1903 (a core-deficient HBV genomic dimer) [31].

### Construction of SV40 LT- HBc 147–183

SV40 LT was amplified from pSVTex (a gift from Dr. Won-Bo Wang) by PCR. The SV40 LT DNA fragment without a stop codon was then subcloned into the pcDNA3.1(+) expression plasmid (the original stop codon of SV40 LT was not included in PCR amplification). An HBc ARD fragment carrying a linker (gly-gly-ser-gly-gly-ser) and a NheI or KpnI restriction site was amplified by PCR.

The HBc DNA fragment, which carried a NheI restriction site and a linker (gly-gly-ser-gly-gly-ser), was cloned to the N terminus of SV40 LT. An HBc DNA fragment, which carried a KpnI restriction site and a linker (gly-gly-ser-gly-gly-ser), was cloned to the C-terminus of SV40 LT. Since the KpnI site encodes glycine and threonine, the total length of the peptide linker between SV40 LT and HBc ARD should be 8 amino acids. DNA sequencing was performed to confirm the identity of the chimeric plasmid construct. The NLS-deficient K128T mutant of SV40 LT was designed as reported previously [21].

### Construction of HIV-1 Rev-HBc 147–183

Plasmid pCMV-Rev (from Dr. Steven Chen) was used as a parental template. Plasmid pCMV-Rev-HBc 147–183 was assembled from the CMV promoter, HIV-1 Rev cDNA and HBc ARD. A KpnI restriction site at the 3′ end of HIV-I Rev cDNA was generated by QuickChange XL Site-Directed Mutagenesis (Stratagene Co.). An HBc ARD fragment carrying a KpnI restriction site and a linker (gly-gly-ser-gly-gly-ser) was amplified by PCR. The HIV-1 NES-defective Rev mutant, containing L78D/E79L mutations [32], [33], was engineered by PCR mutagenesis. The R-to-A mutants of HBc ARD were also engineered by PCR, using mismatched primers as detailed elsewhere [9], [10].

### Cell culture, transfection, and ELISA

Human hepatoma cell line Huh7 and mouse fibroblast cell line NIH-3T3 were maintained in Dulbecco's modified Eagle's medium (DMEM) (Invitrogen) containing 10% fetal bovine serum (Hyclone) at 37°C in the presence of 5% CO_2_. The Lipofectamine2000 (Invitrogen) transfection procedure was according to manufacturer's instructions. The MTT cytotoxicity assay (Promega Co.) and the ELISA of HBsAg (General Biologicals Cooporation, Taiwan) were according to the vendor's protocols.

### Antibodies

In general, primary antibodies were diluted 300-fold to 1,000-fold for IFA experiments, and 1,000-fold to 10,000-fold for Western blot experiments. These primary antibodies include rabbit polyclonal anti-HBc antibodies (Dako or our own preparation from using *E. coli*-expressed HBc 149 of *ayw* subtype as an immunogen). These anti-HBc antibodies can recognize both full-length HBc 183 and truncated HBc 149 of both *ayw* and *adr* subtype origins by IFA and Western blot analysis (data not shown). Other antibodies used in this paper include mouse monoclonal anti-HBc antibody (Hyb-3120, Institute of Immunology, Japan) [34], [35], [36], mouse monoclonal anti-alpha-tubulin antibody (ICON-GeneTex, Taiwan), rabbit polyclonal anti-lamin B1 antibody (ICON-GeneTex, Taiwan), chicken polyclonal anti-Rev (HIV-1) antibody (ICON-GeneTex, Taiwan), mouse and rabbit anti-TAP antibodies (Santa Cruz and ICON-GeneTex, Taiwan), and mouse monoclonal anti-SV40 LT antibody (Abcam). Secondary antibodies were diluted 200-fold for IFA, and 4,000-fold for Western blot, including goat polyclonal anti-mouse horseradish peroxidase (HRP) (Sigma), goat polyclonal anti-rabbit- HRP (Santa Cruz), goat polyclonal anti-rabbit-Rhodamine (Sigma), donkey polyclonal anti-mouse-fluorescein isothiocyanate (Santa Cruz), donkey polyclonal anti-mouse-Rhodamine (Santa Cruz), and goat anti- chicken-IgY-fluorescein isothiocyanate (ICON-GeneTex, Taiwan).

### Immunofluorescence analysis (IFA)

For immunofluorescent staining, cells were cultured on glass coverslips (18×18 mm), and washed with phosphate-buffered saline (PBS) three times before fixation with freshly prepared 4% paraformaldehyde in PBS for 30 minutes at room temperature. Fixed cells were washed three times again with PBS, and permeabilized with 0.1% Triton X-100 in PBS for 30 minutes at room temperature. After the final washing with PBS three times, HBc was stained with a rabbit anti-HBc antibody (Dako), or a mouse monoclonal antibody (Hyb-3210; Institute of Immunology, Japan) which recognizes a capsid conformation-specific epitope [34]. Other primary and secondary antibodies were used as described above. After immunostaining, coverslips were mounted on slides in Prolong Medium containing 4′-6-diamidino-2-phenylindole (DAPI) (Invitrogen). Images were collected using a Zeiss confocal microscope (LSM 510). Pinholes were set to acquire optical sections of <0.8 µm. Images were averaged four times and processed with Zen 2008 Light Edition Zeiss software.

### Fractionation of nucleus and cytoplasm

A ProteoJET Cytoplasmic and Nuclear Protein Extraction Kit (Fermentas, Canada) was used for preparation of the cytoplasmic and nuclear fractions following the vendor's protocol. Briefly, transfected cells were suspended in the cell lysis buffer, vortexed for 10 seconds, and incubated for 10 minutes at 4°C. After the first round of centrifugation at 500g for 7 minutes, the supernatants were subjected to a second round of centrifugation at 20,000g for 15 minutes. Supernatants were collected as the cytoplasmic fractions. The pellets from the first round of centrifugation were rinsed twice with nuclei washing buffer, and resuspended in the nuclei storage buffer. The samples of nuclei in the storage buffer was then added with nuclei lysis reagent, and allowed incubation for 15 minutes at 4°C with repetitive vortexing in every 3 minutes. The resulting nuclear samples were centrifuged at 20,000g for 5 minutes at 4°C, and the supernatants from the centrifugation were collected as nuclear extracts.

### Western blot analysis

Protein samples were prepared from i) total cell lysates, ii) the cytoplasmic fraction, and iii) the nuclear fraction. For the preparation of cytoplasmic and nuclear fractions, the procedure was as described above. For the preparation of total cell lysates, 2×10^5^ Huh7 cells at 48 hours posttransfection were harvested and the cell pellets were lysed in 60 µl of 2× protein lysis buffer (4% SDS, 100mM Tris-HCl [pH6.8], 0.2% bromophenol blue, 5% 2-mercaptoethanol, and 20% glycerol). Approximately 30 µl aliquots of the 400 µl of cytoplasmic fractions from 2×10^6^ cells were mixed with 10 µl 4× protein lysis buffer before loading on the SDS-polyacrylamide gel electrophoresis (PAGE). Similarly, 25 µl aliquots of a total of 50 µl nuclear fractions from 2×10^6^ cells were mixed with 8 µl 4× protein lysis buffer before loading on gel. Protein samples were boiled for 10 minutes before SDS-PAGE and Western blot analysis, and were visualized by enhanced chemilluminescence (Amersham Biosciences).

### Leptomycin B treatment

Approximately 24 hours posttransfection, cells were incubated for 12 hours with leptomycin B (LMB; Sigma) at a final concentration of 60 ng/ml, or left untreated in DMEM containing 10% fetal bovine serum, at 37°C in the presence of 5% CO_2_. Cells were examined by confocal immunofluorescence analysis.

### Heterokaryon assays

Twenty-four hours posttransfection, human Huh7 cells were trypsinized, and 10^6^ Huh7 cells were mixed with 2×10^6^ untransfected mouse NIH-3T3 cells, before seeding on glass coverslips (18×18 mm) in the 6-cm dish. The coculture was maintained at 37°C for 18 hours. Cells were then treated with cycloheximide (Sigma) at 50 µg per ml for 30 minutes prior to fusion. To induce fusion, cells on the coverslips were then covered with 50% (wt/vol) polyethylene glycol (PEG) 8,000 (Sigma) in DMEM for 2 minutes at 37°C. PEG-treated cells were rinsed three times with PBS, and incubated further for 2 hours in DMEM containing 10% fetal bovine serum and cycloheximide at 50 µg per ml at 37°C in the presence of 5% CO_2_ prior to confocal immunofluorescence analysis.

### Homokaryon assays

Huh7 donor cells transfected with pCMV-Rev, pCMV-Rev-HBc ARD, or SV40 LT-HBc ARD chimera, were seeded on coverslips (18×18 mm) in the 6 cm dish, followed by coincubation with an equal number of Huh7 recipient cells transfected with pcDNA-SV40 LT or NES-defective Rev for 18 hours at 37°C in the presence of 5% CO_2_. The rest of procedures were the same as described above in the heterokaryon assay.

### Ethics statement

All animal experiments were conducted under protocols approved by Academia Sinica Institutional Animal Care & Utilization Committee (ASIACUC). Research was conducted in compliance with the principles stated in the *Guide for the Care and Use of Laboratory Animals*, National Research Council, 1996.

### Hydrodynamics-based transfection

BABL/c mice were purchased from the National Laboratory Animal Center (Taipei, Taiwan). The mice were fed with standard chow and water ad libitum. Six- to eight-week old male mice were used in this study. Groups of two BABL/c mice were injected via tail vein with 30 µg of HBV DNA of pCH93091 in a volume of PBS equivalent to 8–10% body weight within 5–7 seconds [37], [38].

### Immunohistochemical (IHC) staining of HBV core antigen

Three days after injection, liver samples were collected and embedded for immunohistochemical staining. The endogenous peroxidase was inhibited by 3% H_2_O_2_/PBS. HBV core antigen was detected by using rabbit polyclonal anti-HBc antibody (Dako). Secondary antibody used in IHC was goat polyclonal anti-rabbit HRP (ICON-GeneTex, Taiwan). Peroxidase stain of brown color was developed by using 3,3′-Diaminobenzidine tetrahydrochloride (DAB, Sigma, USA) as a substrate. The nuclei of liver sections were also counterstained with Mayer's hematoxylin (J.T. Baker, USA).

### Knockdown of TAP by SiRNA

The sequences targeted by chemically synthesized small interfering RNAs (siRNAs, ON-TARGETplus SMARTpool siRNA (Dharmacon, USA)) in transient knockdown experiments were shown as follows : TAP/NXF1 gene (target sequence : GGGAAGUCGUACAGCGAAC; GCGCCAUUCGCGAACGAUU; AAUUGAAGUCUGAGCGGGA; CGAUGAUGAACGCGUUAAU). Briefly, Huh7 cells at about 50% confluence in a 12-well culture dish were transfected with 500ng of plasmid DNA and 50 pmol of siRNA by using Lipofectamine2000 (Invitrogen). Approximately 48 hours post-transfection, cells were harvested and analyzed by IFA or Western blot analysis. To improve the detection sensitivity by Western blot analysis, Huh7 cells were cotransfected with siRNA and a plasmid carrying a puromycin resistance gene (pTRE2pur), followed by incubation with puromycin at 2 µg/ml for 4 days. The majority of untransfected cells were removed by puromycin treatment.

### Immunoprecipitation

The transfected cells (2×10^6^) were collected and lysed with 0.5 ml of immunoprecipitation buffer (25 mM Tris pH7.4, 150 mM NaCl, 0.5% NP-40, 1mM EDTA, 5% glycerol, protease inhibitor cocktail (Roche), and 5mM DTT), and allowed incubation on ice for 20 minutes with occasional mixing. Cell lysates were centrifuged at 13000g and 4°C for 10 minutes. The suspension was transferred to a new tube and mixed with antibodies, and either with or without RNaseA (200µg/mL, Merck). The antigen-antibody complex was allowed incubation at 4°C for 90 minutes with gentle rotation before mixing with protein A sepharose beads (GE Healthcare, USA). The protein A-antibody-antigen complex was further incubated at 4°C for 90 minutes with gentle rotation. The beads were collected by centrifugation at 1000g, 4°C for 20 seconds and washed 5 times in 1 ml immunoprecipitation buffer. The 1× protein lysis buffer was added to release the protein from the beads.

### Construction of a CMV-TAP expression vector

TAP/NXF1 (nuclear RNA export factor 1 isoform 1; NM_006362) cDNA fragment was generated by PCR amplification from a human mammary gland matchmaker cDNA library (Clontech), using the following DNA primers: a forward primer carrying a BamHI restriction site, a kozak sequence (gccacc), and an ATG start codon (5′ ggcggatccgccaccatggcggacgaggggaag 3′); a reverse primer carrying an EcoRI site and a TGA stop codon (5′ gccgaattctcacttcatgaatgccac3′). pcDNA-TAP/NXF1 was constructed by insertion of a ∼1.9kb BamHI - EcoRI fragment containing the TAP/NXF1 cDNA into a pcDNA3.1 (+) expression vector. The TAP/NXF1 cDNA fragment was confirmed by sequencing.

### Southern blot analysis

HBV core-associated DNA from transfected cells was subjected to Southern blot analysis as previously described using a vector free HBV DNA probe [9], [11]. Quantitation of the intensity of the images was done with Image J (National Institutes of Health).

## Results

### Rationale

As described in the Introduction, it remains to be settled where the NLS of HBc is located (Fig. 1; [23], [24], [25]). None of the previous studies was conducted in a system with active viral replication. To revisit this issue, we used here replication-competent HBV DNA and replication-permissive human Huh7 hepatoma cells.

### Alanine scanning of HBc ARD in three different experimental settings using a double-blind protocol

As shown in Fig. 2A, we introduced arginine (R)-to-alanine (A) substitutions at various positions in the ARD of HBc. A total of 15 ARD mutants and the wild type HBV genome were transfected, respectively, into Huh7 cells in three different experimental settings: a) co-transfected with an HBV polymerase (pol) expression plasmid pMT-pol [30]; b) co-transfected with a core-deficient genomic tandem dimer (plasmid 1903) [31]; c) without co-transfection with any other plasmids. Because HBc 136–183 overlaps with the pol open reading frame, mutations in the ARD of HBc could result in concurrent polymerase mutations. To avoid the complication from concurrent polymerase mutations, we provided wild type HBV polymerase *in trans* in the pMT-pol co-transfection experiment. Alternatively, we co-transfected ARD mutants with plasmid 1903, which can produce wild type polymerase, but no HBc protein, due to the ablation of the AUG initiation codon of HBc at nt 1903. To compare the subcellular localizations of HBc in various contexts of HBV DNA replication, we also transfected ARD mutants alone without cotransfection with any other plasmids.

### To distinguish between HBc, precore, and HBeAg

The subcellular localizations of HBc in transfected Huh7 cells were evaluated at 36–48 hours post-transfection by immunofluorescence assay (IFA) using anti-HBc specific antibodies. Because HBc, precore, and HBeAg are immunologically cross reactive, it is important to distinguish them from each other. In the experimental settings with no cotransfection or with pMT-pol cotransfection, no significant amount of HBeAg can be detected by ELISA (data not shown). As mentioned in Materials and Methods, all of these HBc ARD mutants were derived from plasmid pCH93091, which has a tuncated precore open reading frame, and thus is deficient in both precore and HBeAg [29]. Furthermore, in addition to the rabbit polyclonal anti-HBc antibody (Dako, Denmark), we also used a mouse monoclonal antibody Hyb-3120 [36] in the IFA in Fig. 2B. This antibody can recognize specific HBc capsid conformation [34], [35], and fails to recognize HBeAg. Consistent results were obtained using either rabbit anti-HBc antibody (Fig. 2) or mouse Hyb-3120 antibody (data not shown), indicating that it was HBc, rather than precore or HBeAg, that was stained positive in IFA.

To avoid more subjective interpretation of the results by IFA, we coded all the samples before transfection, and scored their subcellular localizations by examining at least 500 HBc-positive cells for each sample in a double-blind manner (Fig. 2B). To confirm the results from IFA, we also performed subcellular fractionation and Western blot analysis using rabbit anti-HBc antibodies (Fig. 2C, Materials and Methods).

### Only one mutant ARD-II+IV exhibited predominant nuclear localization of HBc

To our surprise, only mutant ARD-II+IV, out of a total of 15 mutants, exhibited a strong phenotype of nuclear localization of HBc in approximately 70% of HBc positive cells (Fig. 2A and 2B). In contrast, wild type HBV and most ARD mutants exhibited predominantly cytoplasmic HBc, and nuclear HBc was found in approximately 1% of HBc positive cells. The IFA result of nuclear accumulation of HBc of mutant ARD-II+IV was confirmed by Western blot analysis. Mutant ARD-II+IV exhibited more HBc in the nucleus and less HBc in the cytoplasm (Fig. 2C). Overall, similar conclusions were obtained in all three experimental settings. One caveat here is that when cotransfection was with pMT-pol, we always kept the ratio of plasmids between pMT-pol and HBc ARD mutant less than 1/5 (w/w). Too much polymerase tended to trap more HBc in the cytoplasm and attenuated the phenotype of nuclear accumulation of HBc. On the other hand, without cotransfection with pMT-pol or p1903, the percentage of cells exhibiting the N>C pattern tended to be higher than that of cotransfection experiments. Despite these variations in exact percentages, the general trend of subcellular distribution of HBc remained consistent among different experimental approaches.

As shown in the left panel of Fig. 2D, mutant ARD-I+III and mutant ARD-II+IV, could not replicate by themselves. However, when an intact polymerase (pMT-pol) or a core deficient replicon (p1903) was provided *in trans*, DNA synthesis of both mutants can be partially rescued (middle and right panels, Fig. 2D). The “short DNA phenotype” of mutants ARD I+III and ARD-II+IV is reminiscent of arginine deficiency mutant 164 [11] and mutant 173GG [9]. Due to the “charge imbalance” at the inner surface of HBc capsids, these mutants could be defective in capsid stability, RNA encapsidation, and DNA synthesis [9], [10], [11]. This charge balance hypothesis is under active investigation in another study, and will not be elaborated here. While the short DNA patterns of mutants ARD-I+III and ARD-II+IV were similar to each other (Fig. 2D), their respective HBc distributions were opposite – cytoplasmic predominance (C>N) of the former vs. nuclear predominance (N>C) of the latter (Fig. 2A). In brief, we used three different experimental settings in Fig. 2D (no cotransfection, cotransfection with pMT-pol or cotransfection with core-deficient p1903), and obtained similar results that only the HBc of mutant ARD-II+IV strongly targeted to the nucleus (Fig. 2A).

### Data analysis and interpretations

The motif of NLS is known to contain clustering basic residues [20], [21], [22]. The fact that mutations at ARD-II and ARD-IV can promote nuclear localization of HBc suggests that HBc NLS cannot be located at ARD-II and ARD-IV. Instead, ARD-II and ARD-IV probably are associated with an “anti-NLS signal” inhibitory to HBc nuclear localization. By inference, we hypothesize that a putative NLS of HBc could be associated with ARD-I and ARD-III. Indeed, when mutant ARD-II+IV acquired a third mutation at either ARD-I (i.e., mutant ARD-I+II+IV) or ARD-III (i.e., ARD-II+III+IV), nuclear localization of HBc was almost completely lost (Fig. 2A). This phenomenon suggests that a putative NLS around ARD-I and ARD-III must function in an inter-dependent (co-dependent) or bipartite manner, rather than in an independent manner. According to this scenario, all genotypes with a mutation at ARD-I or ARD-III would be predominantly cytoplasmic (Fig. 2A). Furthermore, since neither single mutant ARD-II nor single mutant ARD-IV is sufficient for nuclear accumulation of HBc (Fig. 2A), we speculate that the anti-NLS signals associated with ARD-II and ARD-IV could each function in a dominant and independent manner.

### Rationale

We wish to test further the hypothesis that ARD-I and ARD-III contain putative NLS, while ARD-II and ARD-IV contain signals inhibitory or opposite to NLS. To this end, we examined the respective functions of HBc ARD-I, -II, -III, and -IV, in a heterologous context of SV40 large T (LT) antigen as a reporter. Unlike the HBV replication system used in Fig. 2, the SV40 LT reporter system has no active HBV replication.

### SV40 large T antigen-HBc ARD reporter

**Figure 3 ppat-1001162-g003:**
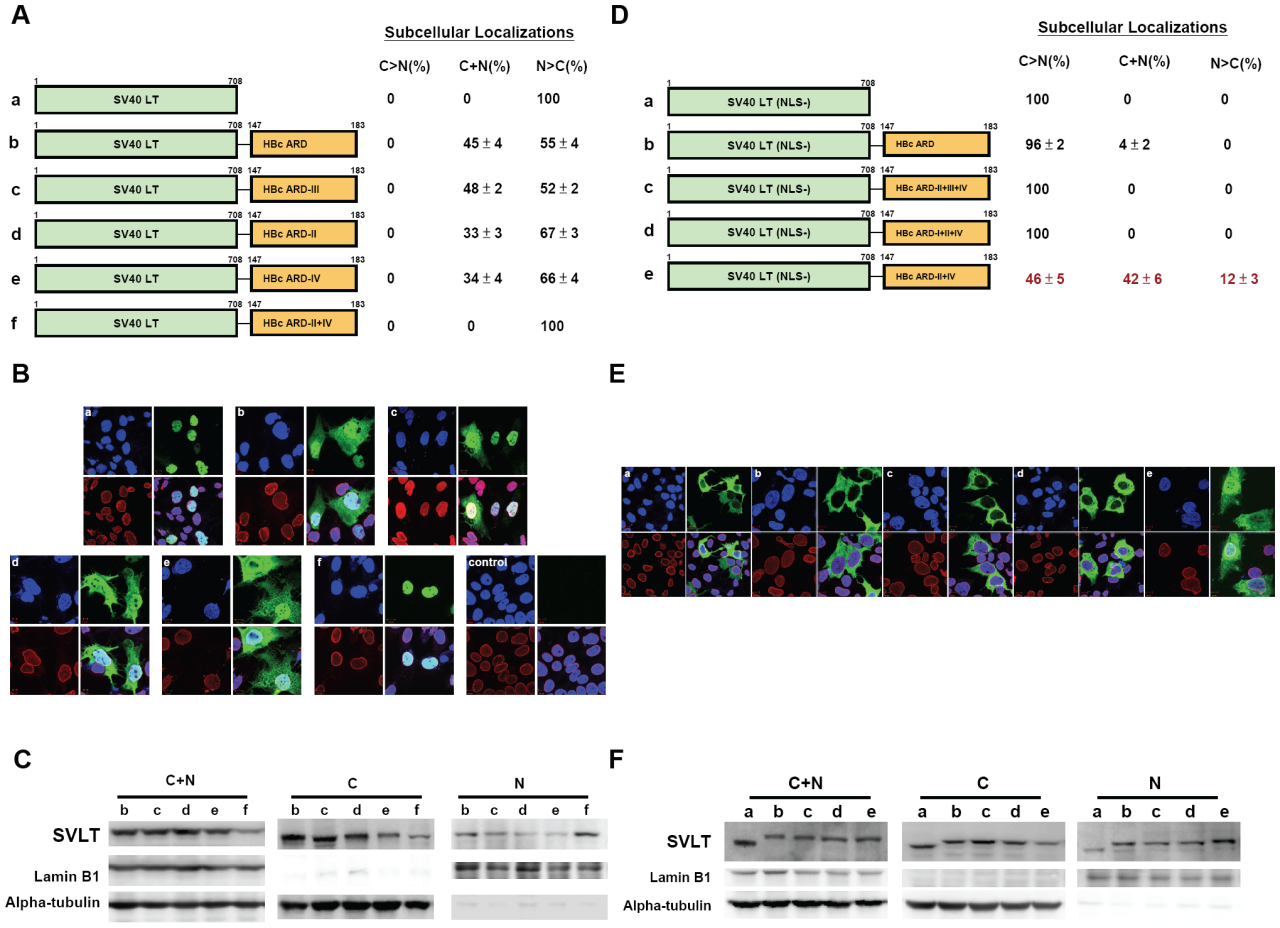
Fig. 3A–3C: Both HBc ARD-II and ARD-IV can independently contribute to cytoplasmic localization of SV40 large T antigen. The definitions of the patterns “N>C”, “C>N”, and “C+N” are as described in the legend to Fig. 2. **Fig. 3B**. Immunofluorescence assay of wild type and various chimeric SV40 LT fused in-frame with wild type or mutant HBc ARD, as illustrated in Fig. 3A. Approximately 300 to 500 cells for each construct were scored for their subcellular localization of SV40 LT. SV40 LT (green), lamin B1 (red), and DAPI (blue). The control sample is from mock transfected Huh7 cells. **Fig. 3C**. Western blot analysis of various chimeric SV40 LT is illustrated in Fig. 3A. **Fig. 3D–3F**: Both HBc ARD-I and ARD-III are required for nuclear localization of NLS-deficient SV40 LT (mutant K128T). When the NLS of SV40 LT was abolished by mutation K128T, 100% of cells displayed cytoplasmic LT (C>N pattern). In contrast, approximately 54% of cells transfected with plasmid SV40LT (K128T)-HBc ARD-II+IV shifted from cytoplasm to nucleus (12% N>C+42% C+N pattern). **Fig. 3E**. Immunofluorescence assay of various chimeric SV40 LT (NLS-defective) as illustrated in Fig. 3D. **Fig. 3F**. Western blot analysis of various chimeric SV40 LT (NLS-defective) as illustrated in Fig. 3D. The weak bandings of SV40LT at lanes b, c, and d in the nuclear fraction are likely from the residual contamination from the cytoplasmic fraction.

SV40 large T antigen contains a potent NLS and is localized predominantly in the nucleus (panel a, Fig. 3B; [20], [21], [22]). We constructed a chimeric reporter by in-frame fusion between wild type SV40 LT and HBc ARD (HBc 147–183). HBc ARD was positioned at either the C-terminus (Fig. 3A) or N-terminus of SV40 LT (data not shown). The results of HBc ARD-SV40 LT are similar to those of SV40 LT-HBc ARD, and therefore, only the latter is shown here. As shown in Fig. 3B, panel b, while approximately 55% of the cells remained to exhibit exclusive nuclear SV40 LT, the other 45% of transfected cells exhibited a “C+N” pattern with SV40 LT present in both nucleus and cytoplasm. A similar pattern of subcellular distribution of SV40 LT was observed when ARD-III contained two arginine-to-alanine (R-to-A) substitutions (Fig. 3B, panel c). Interestingly, when R-to-A mutations were introduced into both ARD-II and ARD-IV of HBc, SV40 LT was again found exclusively in the nucleus in 100% of transfected cells (Fig. 3B, panel f). The redistribution of SV40 LT from cytoplasm back to nucleus was not 100% when mutations were introduced into ARD-II alone, or ARD-IV alone (Fig. 3B, panel d and e). These results here lend a strong support for the hypothesis that ARD-II and ARD-IV are associated with anti-NLS like activity. Furthermore, such anti-NLS like activities of ARD-II and ARD-IV appeared to function in a dominant and independent manner (i.e., either one alone is sufficient to inhibit or override NLS). To confirm the results of IFA in Fig. 3B, we performed fractionation and Western blot analysis (Fig. 3C). Indeed, the signal of SV40 LT in the nucleus is relatively stronger for SV40 LT-HBc ARD II+IV (lane f of nuclear fraction, Fig. 3C) than other chimeras (SV40 LT-HBc ARD-II and SV40 LT-HBc ARD-IV in lanes d and e, Fig. 3C). This can even be better appreciated when considering the banding intensity of the total cell lysates (C+N) of mutant ARD-II+IV is relatively weaker (lane f of C+N panel, Fig. 3C). The weaker signal of the steady state level of the chimeric protein of SV40 LT-HBc ARD-II+IV could result from a difference in antibody recognition or protein half lives. For the detection of the phenotype of nuclear accumulation of HBc (N>C pattern), IFA in general appeared to be a more sensitive method than Western blot analysis.

### NLS-defective SV40 large T antigen-HBc ARD reporter

As shown in Fig. 2, ARD-I and ARD-III are likely to be associated with a putative NLS. To test further this association, we ablated the NLS of SV40 LT by a K128T mutation (Fig. 3D; [21]). As shown in the IFA of panels a and b, Fig. 3E, NLS-defective SV40 LT is almost exclusively cytoplasmic, with or without a fused HBc ARD. However, when the NLS-defective SV40 LT is fused in-frame with HBc ARD with intact ARD-I and ARD-III as well as mutant ARD-II and ARD-IV, this chimeric SV40 LT was relocalized to nucleus in 54% of transfected cells (12% N>C and 42% C+N; Fig. 3D). This result suggests that ARD-I and ARD-III could be associated with a less potent NLS, which can partially substitute for a more potent NLS native to SV40 LT, albeit the rescue is not 100% (Fig. 3E, panel e). Alternatively, the relatively weaker NLS of HBc could be related to its serine phosphorylation at HBc ARD (unpublished results). Furthermore, an intact ARD-I alone, or an intact ARD-III alone, is not sufficient for the NLS-like activity (panels c and d, Fig. 3D and 3E), indicating that both ARD-I and ARD-III are required for the NLS-like activity. Most likely, the NLS activity of ARD-I and ARD-III must act together, co-dependently or synergistically. Western blot analysis confirmed the results by IFA that a stronger banding signal of the chimera protein of NLS-defective SV40 LT-HBc ARD-II+IV was accumulated in the nucleus (lane e, nuclear fraction, Fig. 3F).

### HBc ARD can behave like a cytoplasmic retention signal (CRS) by homokaryon and heterokaryon analyses

**Figure 4 ppat-1001162-g004:**
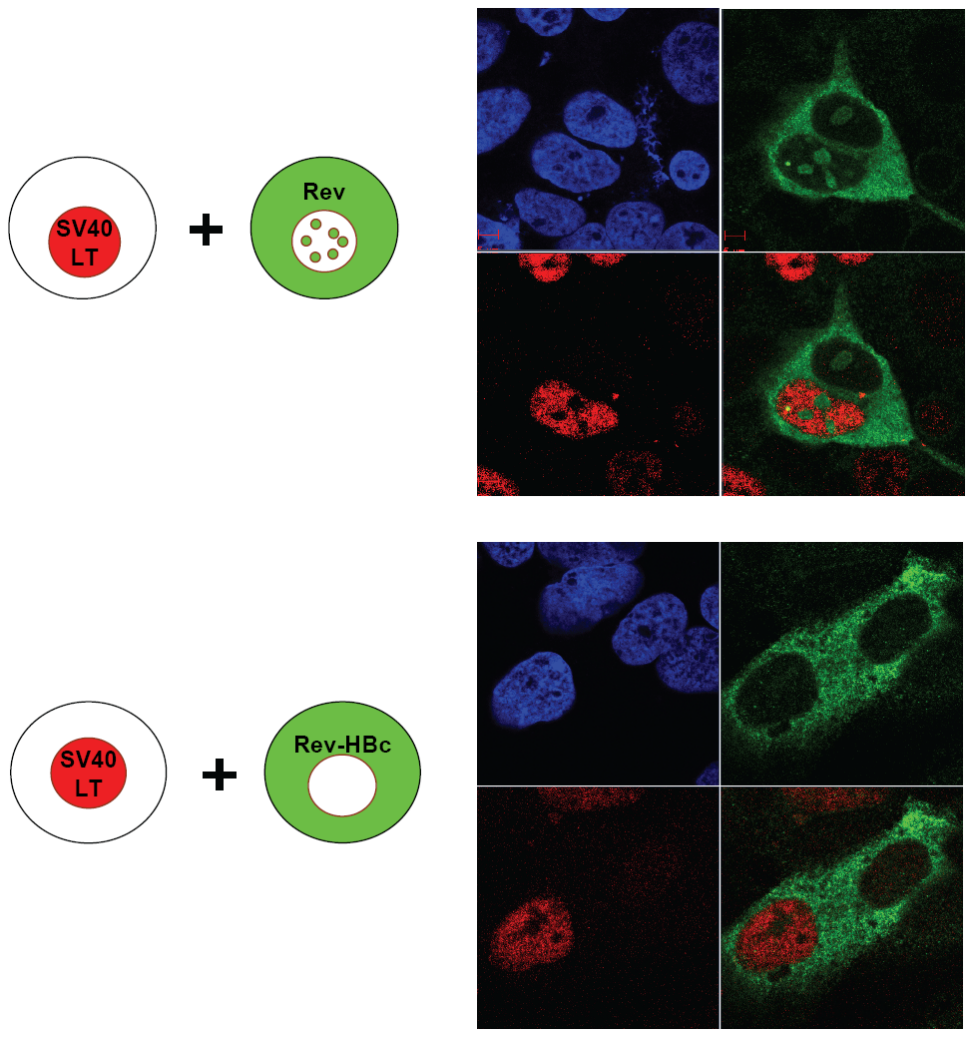
Homokaryon analysis demonstrated that HBc ARD domain (HBc 147–183) can act like a cytoplasmic retention signal (CRS) by inhibiting nuclear import of Rev of Huh7 cells. *Upper panel*, Human Huh7 cells were transfected with pCMV-Rev (green) before fusion with Huh7 cells expressing SV40 LT (red). *Lower panel*, Human Huh7 cells were transfected with pCMV-Rev-HBc ARD (green) before fusion with Huh7 cells expressing SV40 LT (red). DAPI (blue). No nuclear Rev signal was detected.

To analyze further the anti-NLS activity of HBc ARD, we tested by homokaryon analysis (Fig. 4), whether such an anti-NLS activity could indeed function like a so-called cytoplasmic retention signal (CRS), as reported for ApoBEC3G and cyclin B1 in literature [39], [40]. We transfected Huh7 cells with pCMV-Rev, before fusion with Huh7 cells transfected with SV40 LT. By IFA (*upper panel*, Fig. 4), we observed nuclear localization of Rev (green color) in Huh7 cell nuclei with and without expressing SV40 LT (red color). This result indicates that nuclear import of Rev into Huh7 cell nuclei expressing SV40 LT can occur efficiently in the homokaryons. In contrast, no co-localization of Rev and SV40 LT proteins was observed, when Rev was fused in-frame with HBc 147–183 (*lower panel*). These results in Fig. 4 reinforced the previous conclusion that HBc ARD can inhibit or override the NLS-mediated nuclear import, suggesting that HBc ARD could behave like a CRS [39], [40]. This result was also confirmed by heterokaryon analysis using human Huh7 cells and mouse NIH3T3 cells (Supporting Information Figure S1).

### HBc ARD can behave like a nuclear export signal (NES) by homokaryon and heterokaryon analyses

#### Rationale

We detected anti-NLS or CRS-like activity associated with HBc ARD-II and ARD-IV (Fig. 2–4). It is theoretically possible that this phenomenon associated with HBc ARD could reflect the existence of a potent nuclear export signal (NES) [41], [42] in ARD-II and ARD-IV. For example, if the strength of an NES can outweigh that of an NLS within the same protein molecule, then this protein at a steady state could appear to reside in the cytoplasm, even though it could actually be shuttling rapidly between the nucleus and cytoplasm in a dynamic manner.

**Figure 5 ppat-1001162-g005:**
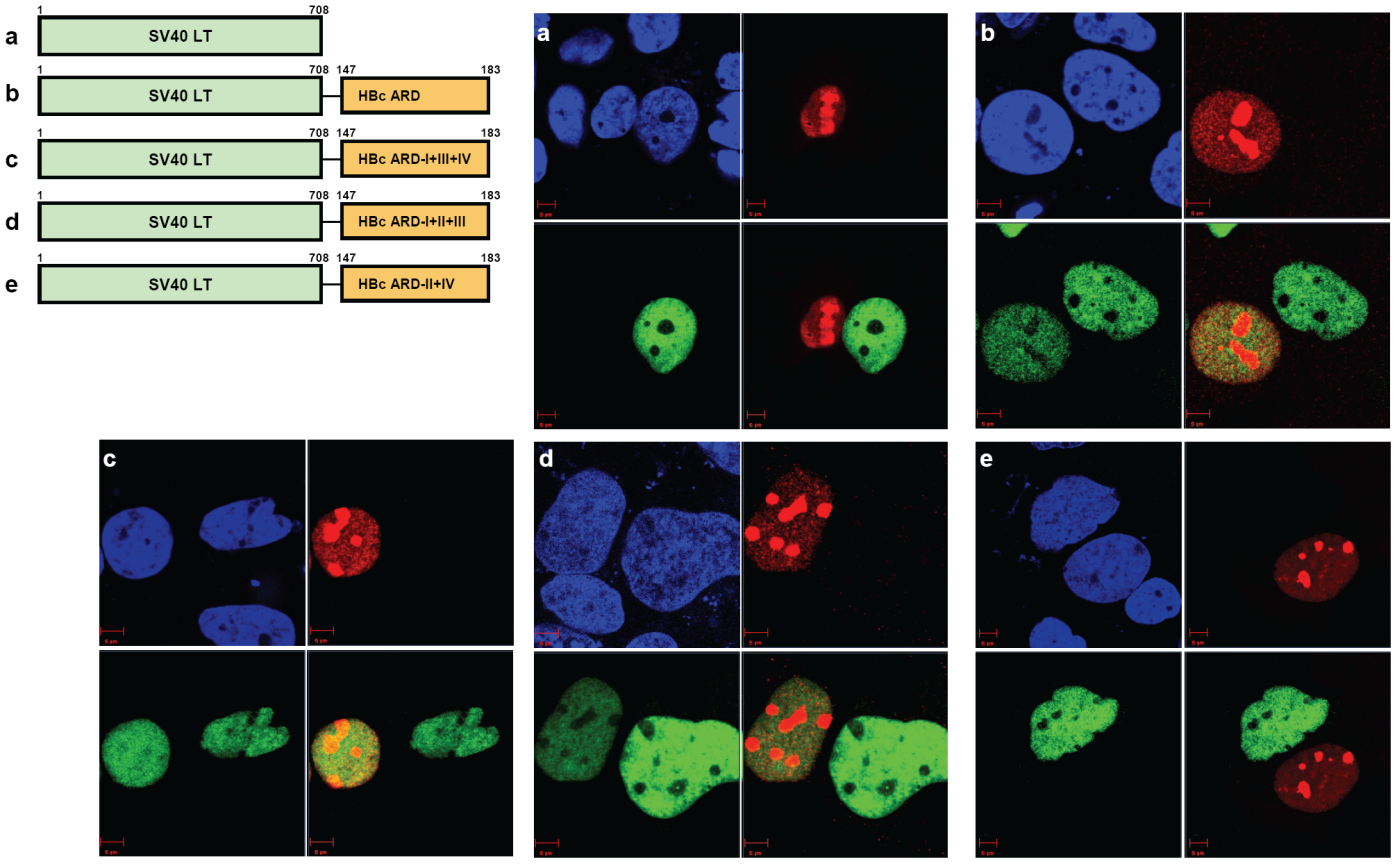
Homokaryon analysis revealed the existence of two independent NES-like signals in HBc ARD-II and ARD-IV. Huh7 donor cells transfected with various versions of SV40LT-HBc chimera (green) were fused with Huh7 recipient cells transfected with NES-deficient Rev (red). DAPI (blue). a) SV40 large T antigen is localized exclusively to the nucleus. There was no transport of SV40 LT from donor to recipient nuclei. b) Chimeric protein of SV40 LT-HBc appeared to shuttle from donor to recipient nuclei. c) Mutant HBc ARD-I+III+IV contains an intact subdomain ARD-II. When this mutant ARD-I+III+IV was fused with SV40 LT, shuttling appeared to occur via the intact ARD-II. d) Mutant HBc ARD-I+II+III contains an intact subdomain ARD-IV. When this mutant ARD-I+II+III was fused with SV40 LT, shuttling appeared to occur via the intact ARD-IV. e) No apparent shuttling was observed for mutant ARD-II+IV, when both ARD-II and ARD-IV subdomains were inactivated. This consistent negative result was based on the examination of a total of 50–60 homokaryons in each experiment, and was controlled by the positive results in panels 5b, 5c, and 5d. Similar results were obtained using a heterokaryon analysis (Supporting Information Figure S4).

We performed homokaryon analysis by cell-cell fusion between Huh7 donor cells transfected with SV40 LT and Huh7 recipient cells transfected with NES-deficient Rev. Both SV40 LT and NES-deficient Rev are localized exclusively to the nucleus. We had examined at least a total of 60 homokaryons for each chimeric construct in Fig. 5. We observed no transport of SV40 LT (green) to nuclei expressing NES-deficient Rev (red), and vice versa (panel a, Fig. 5). In contrast, when SV40 LT was fused with HBc ARD, we detected co-localization of SV40 LT (green) and NES-deficient Rev (red) in the same nucleus (yellow), suggesting the transport of SV40 LT-HBc ARD chimeric protein from one donor nucleus into another recipient nucleus (panel b, Fig. 5; also cf Supporting Information Figures S2 and S3). We could observe this kind of binucleated pattern (one green nucleus and one yellow nucleus) in approximately 50% of homokaryons. A similar phenomenon of shuttling between nuclei can be observed when SV40 LT was fused with HBc ARD-I+III+IV (i.e., ARD-II is structurally intact) or HBc ARD-I+II+III (i.e., ARD-IV is structurally intact) (panels c and d, Fig. 5). However, when SV40 LT was fused with HBc ARD-II+IV, no yellow nuclei were ever detected, indicating the lack of nucleocytoplasmic transport between nuclei (panel e, Fig. 5; also cf Supporting Information Figure S4). Similar results were obtained using a heterokaryon analysis (Supporting Information Figures S2, S3 and S4). An alternative interpretation of Fig. 5e is that the donor and recipient cells are adjacent to each other, but not truly fused into a homokaryon, and thus shuttling between these two nuclei separated by their plasma membranes was not possible. This interpretation is unlikely since we examined a total of 50–60 “homokaryons” in each experiment, and scored zero shuttling by SV40 LT-HBc ARD-II+IV in several independent experiments. It should be noted that the negative results in Fig. 5e was controlled by the positive results in Fig. 5b, 5c, and 5d.

Overall, these results of homokaryon and heterokaryon analyses strongly suggest the existence of two independent NES-like signals associated with ARD-II and ARD-IV, respectively. The combined results of Fig. 4 and 5 indicate that the same HBc ARD in different contexts (SV40 LT vs. Rev) could behave differently (NES vs. CRS) (Supporting Information Figure S3). We speculate here that the relative strength between an NLS and an NES could determine the trafficking behavior and steady state residence of a protein.

### HBc ARD is leptomycin B resistant

**Figure 6:**
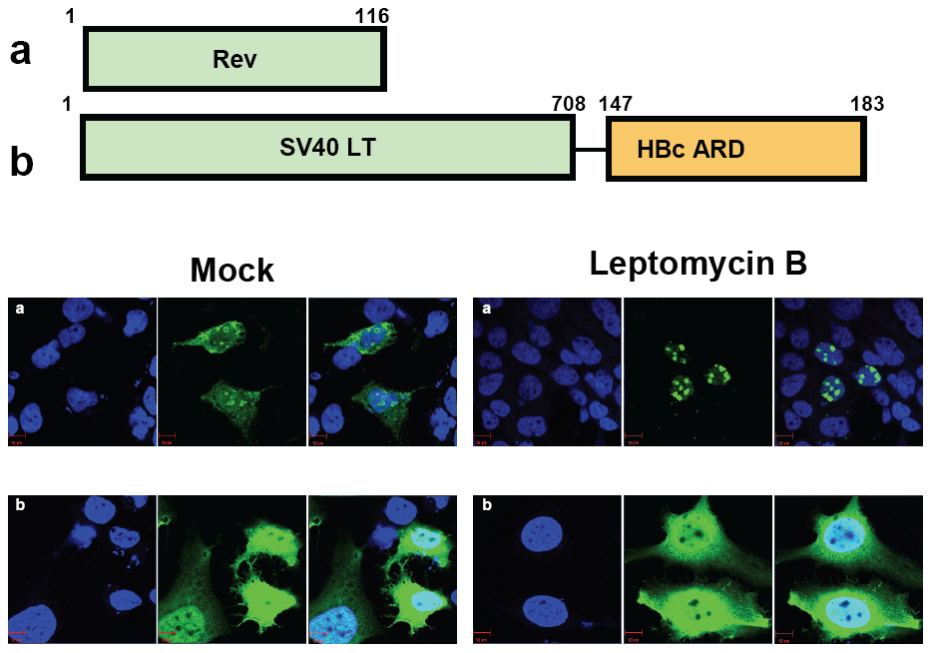
Wild type HBc ARD is leptomycin B resistant, suggesting that HBc ARD does not contain a CRM-1 dependent NES. Immunofluorescence assay of Rev or SV40 LT-HBc ARD signals was performed using transfected Huh7 cells with or without leptomycin B treatment. Rev and SV40 LT (green) and DAPI (blue). a) Wild type Rev in Huh7 cells exhibited both nuclear and cytoplasmic Rev (left panel, mock). Upon treatment with leptomycin B, cytoplasmic Rev was lost (right panel). b) When SV40 LT was fused with wild type HBc ARD, SV40 LT was distributed in both cytoplasm and nucleus in approximately 50% of transfected Huh7 cells (Fig. 3A). The percentage of cells exhibiting this C+N pattern of SV40 LT was not affected upon treatment with leptomycin B for 12 hrs (data not shown).

The prototype NES is known to be sensitive to leptomycin B (LMB), and is often rich in leucine residues [43], [44]. Upon treatment with leptomycin B, cytoplasmic Rev disappeared in Huh7 cells transfected with wild type Rev, and all the Rev signals became localized in the nuclei by IFA (panel a, Fig. 6). However, in the case of HBc ARD, there is no leucine rich motif. In contrast to Rev, SV40 LT is known to have a potent NLS, but without any NES. Therefore, the subcellular localization of SV40 LT should be independent from LMB treatment (data not shown). When SV40 LT was fused in-frame with HBc ARD, approximately 50% of transfected cells exhibited both nuclear and cytoplasmic protein of SV40 LT-HBc ARD chimera (C+N pattern). Upon treatment with LMB, no significant change in the percentage of cells exhibiting a C+N pattern, or an N>C pattern, was ever noted (panel b, Fig. 6). This result indicates that the NES-like activity of HBc ARD is leptomycin resistant and Crm-1 independent [43], [44].

### Specific physical and functional interaction between HBc ARD and TAP

Our studies here strongly suggest that ARD-II and ARD-IV could behave like a CRS or NES (Fig. 2–5). In contrast to the prototypic Crm-1 dependent and leucine-rich NES motif, ARD-II and ARD-IV contain arginine-rich NES-like motifs. Recently, a cellular factor TAP has been reported to be essential for the nuclear export of mRNA and proteins by its physical association with an NES rich in arginine residues [27], [45].

### Physical interaction between TAP and HBc ARD

**Figure 7:**
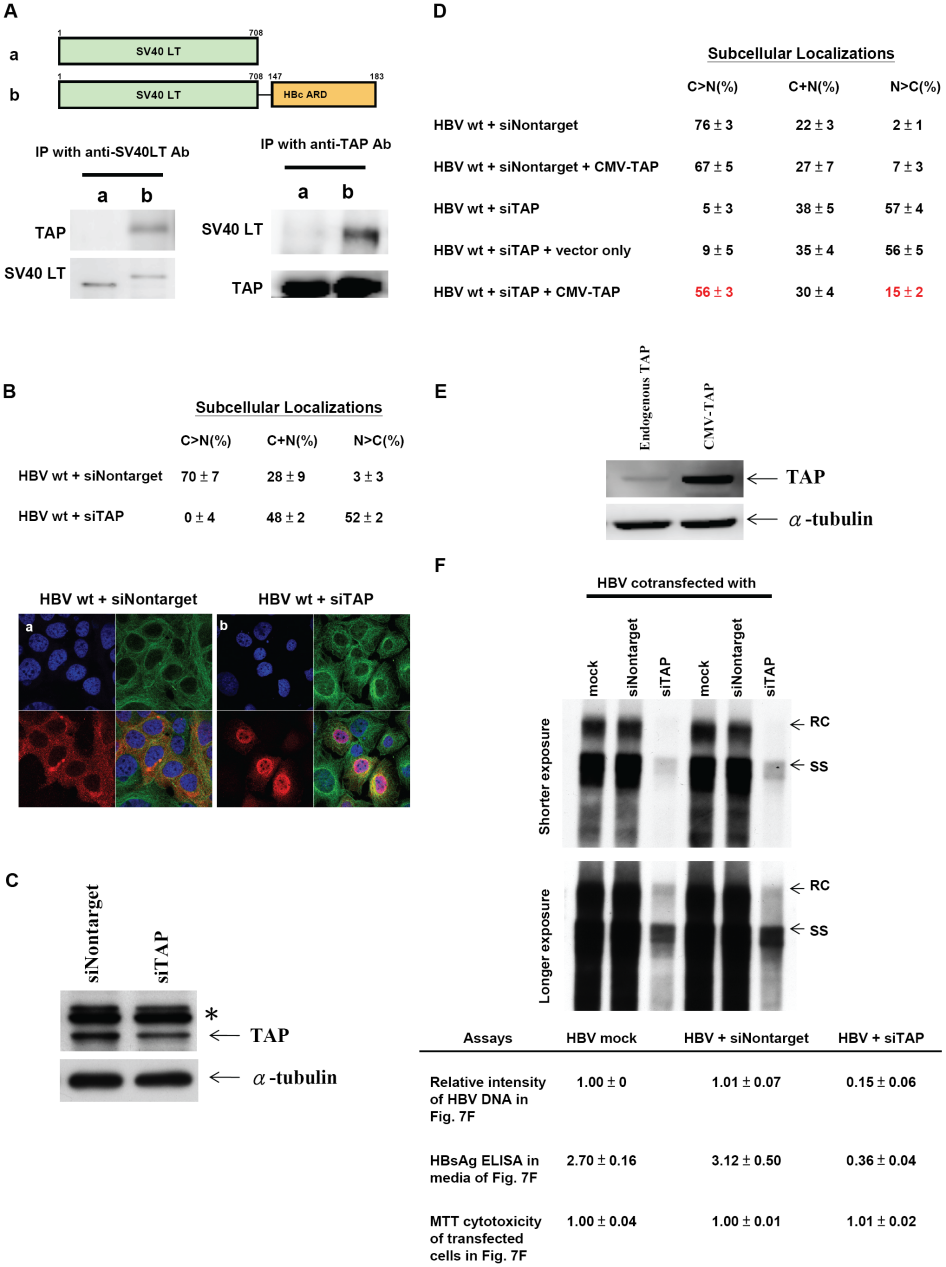
Specific physical and functional interactions between a cellular TAP protein and HBc ARD were shown by experiments of co-immunoprecipitation, si-RNA treatment, and cotransfection with a CMV-TAP expression vector. **Fig. 7A**. Huh7 cells were transfected with expression vectors of a) SV40 LT, or b) SV40 LT-HBc ARD chimera. Transfected cell lysates were immunoprecipitated (IP) with anti-SV40 LT antibody. Equal amounts of immunoprecipitated cell lysates from a) or b) were loaded on SDS-PAGE, respectively, followed by Western blot analysis using anti-TAP or anti-SV40 LT antibodies. The 66 kDa TAP protein can be detected only in cell lysates transfected with SV40 LT-HBc ARD, but not transfected with wild type SV40 LT, indicating that TAP could bind to HBc ARD. This result was confirmed in a reciprocal experiment using an anti-TAP antibody for IP. **Fig. 7B**. Knockdown of endogenous TAP protein by siRNA against TAP (*panel b*) resulted in a more than 17-fold increase of nuclear accumulation of wild type HBc (N>C pattern shifted from 3% to 52%). In contrast, control siRNA (Nontarget) (*panel a*) did not affect the subcellular localization pattern of HBc. HBc (red), α-tubulin (green) and DAPI (blue). **Fig. 7C**. Treatment of Huh7 cells with TAP-specific siRNA resulted in appreciable reduction of TAP protein by Western blot analysis using anti-TAP antibody. * Non-specific bands served as an internal control. (cf Materials and Methods for detail). **Fig. 7D**. The nuclear predominant phenotype of wild type HBc in Huh7 cells treated with siTAP can be reverted efficiently to a cytoplasmic predominant phenotype by cotransfection with a CMV-TAP expression vector. **Fig. 7E**. Western blot analysis of TAP protein in Huh7 cells transfected with a vector only or a plasmid CMV-TAP. **Fig. 7F**. Upon treatment with siTAP, wild type HBV DNA synthesis in Huh7 cells was reduced approximately 7-fold by Southern blot analysis. RC: relaxed circle DNA, SS: single-strand DNA. While there was no difference in the results of MTT cytotoxicity assay using media from cell culture with or without siTAP treatment, the HBsAg level by ELISA was reduced by approximately 10-fold with siTAP treatment. The averaged values of DNA intensity, MTT assay, and HBsAg assay were processed from four independent experiments (Materials and Methods).

To test the possibility that the NES-like activity of ARD-II and ARD-IV could be TAP dependent, we examined the potential interaction between TAP and HBc ARD by immunoprecipitation and Western blot analysis (Fig. 7A). Our initial attempt to look for the association between wild type full-length HBc and TAP was not successful (data not shown), probably due to the fact that the ARD tail of wild type HBc tends to be buried inside the capsid interior [46]. As an alternative approach, Huh7 cells were transfected with either plasmid SV40 LT or plasmid SV40 LT-HBc ARD, and cell lysates were immunoprecipitated with an antibody specific for SV40 LT. TAP protein, as visualized by Western blot analysis, can be coimmunoprecipitated from cell lysates transfected by plasmid SV40 LT-HBc ARD, but not by plasmid SV40 LT. In a reciprocal experiment, similar results were obtained when anti-TAP antibody was used for coimmunoprecipitation (Fig. 7A). Furthermore, the physical association between SV40 LT-HBc ARD and TAP proteins was resistant to RNase treatment (data not shown). These results suggest that the exposed HBc ARD and TAP can bind directly to each other *in vivo*.

### SiRNA specific for TAP/NXF1 can induce nuclear accumulation of wild type HBc and significantly reduce HBV DNA synthesis

In addition to the physical interaction between TAP and HBc ARD, functional interaction between these two proteins was demonstrated by TAP-specific siRNA treatment and IFA. As shown in panel a, Fig. 7B, upon treatment with a control siRNA Nontarget, more than 70% of transfected Huh7 cells exhibited HBc signals predominant in the cytoplasm by IFA, and only about 3% of cells exhibited nuclear dominant HBc. In sharp contrast, upon treatment with a TAP-specific siRNA, only approximately 0–4% of cells exhibited HBc predominant in the cytoplasm, and approximately 52% of cells exhibited the nuclear dominant HBc pattern (panel b, Fig. 7B). We noted that whenever the signal of nuclear HBc was stronger, a rim-like structure around the nuclear periphery was observed. We speculated that HBc was accumulated near NPC, and unable to exit due to the lack of TAP. The reduction of the endogenous TAP protein upon treatment with siTAP can be demonstrated by Western blot analysis (Fig. 7C). Because of the limitation of the transfection efficiency of Huh7 cells, the reduction of the TAP protein level in transfected cells may not be easily detectable in the total cell lysates including both transfected and untransfected cell populations. To circumvent this problem, siTAP was cotransfected with a plasmid carrying puromycin resistance. Treatment of transfected Huh7 cells with puromycin after transfection indeed helped to enrich the transfected cell population, and improved the detection sensitivity by Western blot analysis using a rabbit anti-TAP antibody (Fig. 7C). Furthermore, nuclear accumulation of HBc induced by siTAP can be reverted to a cytoplasmic dominant pattern by cotransfection with a CMV-TAP expression vector (Fig. 7D). This expression vector produced a TAP specific protein recognized by anti-TAP antibody in Western blot analysis (Fig. 7E). These results indicate that there is no off-target effect of siTAP on HBc nuclear accumulation. In Rev transfected Huh7 cells, no difference in Rev distribution by IFA was observed between treatments with siTAP vs. siNontarget (Supporting Information Figure S5), indicating that siTAP has no significant effect on Crm-1 dependent nuclear export of Rev. Most importantly, this result also argues against the possibility that the nuclear accumulation of HBc induced by siTAP treatment could be the consequence of a non-specific effect of siTAP.

As mentioned in the Introduction, cytoplasmic predominant pattern of HBc distribution is often associated with disease activity and more active viral replication [14], [15], [16], [17], [18]. We therefore asked if siTAP treatment, which resulted in HBc accumulation in the nucleus, could have any effect on HBV replication. As shown in Fig. 7F, siTAP reduced HBV DNA synthesis by at least 7-fold, as well as the HBsAg level in the media by approximately 10-fold, relative to mock or siNontarget controls. Not only HBV DNA synthesis can be reduced by siTAP in a dose dependent manner, but also the reduction of HBV DNA can be significantly rescued by cotransfection with a CMV-TAP (data not shown). In contrast, no apparent effect from siTAP on cytotoxicity was detected by MTT assay (Fig. 7F). Taken together, a drastic shift of wild type HBc from cytoplasm to nucleus can be triggered by the reduction of TAP protein. This result supports that nuclear export of HBc could be mediated through TAP, and nuclear accumulation of HBc is associated with less active viral replication [14], [15], [16], [17], [18]. One interpretation of the Southern results is that nuclear accumulation of HBc is a direct cause of reduced viral DNA synthesis. An alternative explanation is that TAP knock down could somehow inhibit viral DNA synthesis independent of HBc nuclear retention. For example, if the reduction of TAP could lead to the reduction of mRNA export of liver transcription factors, then the reduction of viral DNA synthesis could primarily originate from a reduced level of viral transcription.

### Hydrodynamic delivery in the mouse animal model

In addition to Huh7 cell culture, we used the hydrodynamic delivery system to introduce HBV DNA into mouse hepatocytes [37], [38]. Sectioned liver samples collected on day 3 post-injection were fixed and stained immunohistochemically, and the subcellular distribution of HBc was scored under microscope in a double blind manner.

**Figure 8:**
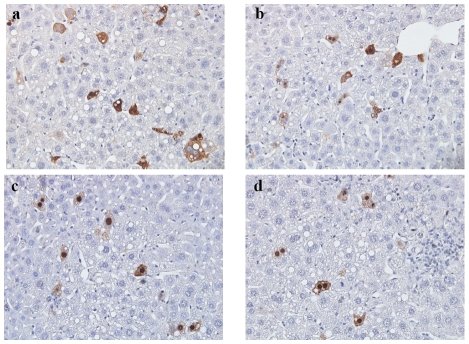
*In vivo* subcellular localization of core proteins encoded by mutants HBc ARD-I+III and ARD-II+IV was examined by immunohistochemistry staining and hydrodynamic delivery. The liver tissue was collected 3 days post injection with the following plasmids: a) wild type HBc was mainly distributed in the cytoplasm; b) HBc of mutant ARD-I+III was also mainly distributed in the cytoplasm; a central vein-like structure is shown near the upper right corner. c & d) HBc of mutant ARD-II+IV was mainly distributed in the nucleus; Inflammatory lymphocyte infiltration was noted. Magnification, ×40. The relative frequencies of different patterns of subcellular localization of HBc were scored in a double blind manner (Table 1).

As shown in Table 1, approximately 50% of hepatocytes transfected with wild type HBc exhibited the C+N pattern on day 3 post-injection. The remaining 50% of transfected cells were about equally split between the C>N pattern and the N>C pattern. An example of wild type HBc exhibiting the C>N pattern and C+N pattern on day 3 post-injection is shown in Fig. 8a. Plasmids of mutants ARD-II+IV and ARD-I+III are based on an HBV replicon pCH93091 [29]. Both ARD mutants are replication defective (Fig. 2D). In the sectioned liver, mutant HBc ARD-I+III exhibited a subcellular distribution profile similar to that of wild type HBc, with a predominant C+N pattern, and a slightly reduced percentage of the N>C pattern (Table 1). An example of HBc subcellular distribution of mutant ARD-I+III exhibiting the C+N pattern and C>N pattern is shown in Fig. 8b. In contrast, mutant HBc ARD-II+IV exhibited a nuclear predominant HBc pattern (N>C, 45.77–64.07%, Table 1) (Fig. 8c and 8d) and the lowest percentage of C>N pattern (7.30–8.56%). Taken together, the results from the Huh7 cells in culture did not seem to parallel very well with that of the hydrodynamic approach using the mouse model (Fig. 2A vs. Table 1). This is particularly true considering the N>C pattern of wild type HBc in culture is only around 1% (Fig. 2A), while the N>C pattern of wild type HBc *in vivo* using the hydrodynamic approach is around 26% (Table 1). Despite this difference in the percentages of subcellular distribution patterns of wild type HBc, the overall trends of the distribution patterns of HBc ARD mutants are entirely consistent between the cell culture and the mouse model (for the N>C pattern, HBc ARD-I+III < wild type HBc≪HBc ARD-II+IV). The results from the hydrodynamic studies (Table 1) lend support for the cell culture studies that ARD-II and ARD-IV are associated with anti-NLS/CRS/NES activity, while ARD-I and ARD-III are associated with NLS activity (Fig. 2–6).

**Table 1:** Comparisons of the Subcellular Distribution of HBV Core Protein in the Liver of Hydrodynamically Injected BALB/c Mice by Immunohistochemical Staining*.

HBc genotypes	total number of cells counted	C>N (%)	C+N (%)	N>C (%)
WT	443	25.06	48.53	26.41
	864	23.38	50.00	26.62
Mutant ARD -I+III	808	21.29	61.88	16.83
	573	29.32	45.90	24.78
Mutant ARD -II+IV	526	8.56	27.38	64.07
	603	7.30	46.93	45.77

*For each HBc genotype, two BALB/c mice were injected intravenously, and HBc expression was examined in a double blind manner in sectioned liver samples on day 3 post-injection by IHC using anti-HBc antibody. Definitions of C>N, C+N, and N>C patterns are as described in the legend to Fig. 2.

## Discussion

### Comparisons between our current study and previous reports in literature

**Figure 9:**
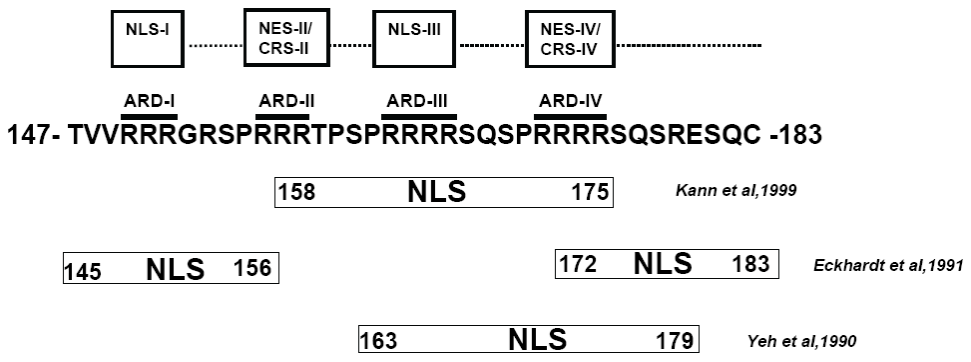
A summary of novel trafficking signals of HBc core protein and particles identified in this study vs. those reported previously [23], [24], [25]. The boundaries between NLS and NES/CRS, which remain to be defined in the future, are shown by dotted lines.

As shown in Fig. 9, the results from this study are very different from those in the literature in two major ways: 1) We identified two independent CRS/NES-like elements in HBc ARD-II and ARD-IV which have not so far been reported. 2) We identified two co-dependent NLS-I and NLS-III which have not been reported previously [23], [24], [25]. In the report by Kann et al. [25], the study of nuclear import was based on a cell-free nuclei system without cytoplasm. Such a system was designed for the assay of nuclear import, but not for nuclear export. The reports by Yeh et al. [23] and Eckhardt et al. [24] were based on the cell culture system. However these reports were published in 1990 and 1991, at a time before NES was better known [41], [42]. Naturally, earlier studies were focused only on NLS, but not NES. As for the differences in the mapped positions of HBc NLS, they are likely due to the differences in the respective assay systems, e.g., *in vitro* [25] vs. *in vivo* [23], [24], and stable [24] vs. transient [23] transfections. Part of the approach by Eckhardt et al (1991) relied on stable transfection of mouse NIH3T3 cells using an expression vector of intact or truncated HBc. Deletion at the HBc ARD could include both arginines and adjacent serines. It is well known that serine phosphorylation could influence the trafficking behavior of HBc [25], [47], [48]. In our approach (Fig. 2–Fig. 8), serine residues and the total length of HBc were both preserved in the native context. Eckhardt et al. [24] reported that a single copy of arginine rich motif (ARD-I or ARD-IV) is sufficient for nuclear localization of HBc. In contrast, Yeh et al. [23] reported that at least two copies of arginine rich motif are required for nuclear localization of HBc. Our current finding supports the notion that two copies (both NLS-I and NLS-III) are required for efficient nuclear localization of HBc (Fig. 9).

Recently, Weigand et al. [49] reported that the HBc ARD contains no NLS using a GFP-HBc chimeric reporter assay. This is in contradiction with existing literature and our current findings of NLS-I and NLS-III. Furthermore, Weigand et al. [49] reported that a capsid-assembly dependent NES is present in HBc ARD. In contrast, the NES/CRS identified in our Rev or SV40 LT chimeric reporter systems are without capsid assembly (Fig. 3 and data not shown). It is less clear what an NES can be used for, if no NLS can be used for nuclear import by a cytoplasmic protein. It might also be useful to distinguish the possibility of co-assembly of GFP-HBc and wild type HBc from that of co-packaging of GFP-HBc inside the capsids of wild type HBc [49].

### The paradox of functional diversity with structural identity

It is intriguing that the amino acid sequences of these four ARD subdomains are strikingly similar, yet their associated functions could be so different or even opposite (NLS vs. NES/CRS). It is possible that their functional difference could be related to the following: 1) their structural differences in amino acid sequences at the boundary regions of these four ARD subdomains (Fig. 9). For example, the C-terminal boundary of NES-IV could include sequences that are absent in NLS-III. 2) HBc is a phosphoprotein [50] and phosphorylation of serine residues at the boundaries has been shown to influence nuclear transport of HBc (e.g., [25], [47], [48]), presumably by modulating the activities of NLS and NES. Conversely, mutations at arginine residues could affect phosphorylation of adjacent serines. It is possible that posttranslational modification might render ARD-III or ARD–IV exerting different functions, despite the same amino acid sequences. 3) The same stretch of amino acid sequences could be folded into different 3D structures or positioned in different contexts of 3D structures. An example of the dual topology of the preS1 polypeptide of HBV surface antigen can be cited here [51].

### Hierarchy among different trafficking signals

In the transient transfection system, wild type HBc is predominantly cytoplasmic (Fig. 2). Therefore, HBc NES/CRS appeared to be dominant over NLS, at least in the transient transfection assay. However, it is less clear to us at present concerning the relative strength between NES-II/CRS-II and NES-IV/CRS-IV. As for the relative strength between NLS-I and NLS-III, NLS-III appeared to be stronger than NLS-I. When NLS-I was mutated in mutant ARD- I+II+IV, nuclear HBc could still be detected in 4–10% of transfected Huh7 cells. Conversely, when NLS-III was mutated in mutant ARD-II+III+IV, we could not detect any nuclear HBc (Fig. 2A).

### NLS of HBc protein vs. NLS of HBc particles

The C-terminus of HBc has been mapped to the capsid interior in icosahedral particles [46]. It is therefore unclear how NLS and NES/CRS in the capsid interior can be recognized by the cellular machinery involved in nuclear import. Previously, we proposed a “charge balance hypothesis” of HBc capsid stability and assembly (also coined “balanced electrostatic interaction hypothesis”) [9], [10], [11]. Alterations of capsid conformation can be triggered by charge imbalance in the capsid inner surface. When the plus-strand DNA synthesis begins to accumulate more and more negative charge from the elongating phosphodiester backbone, it is postulated that capsid instability would gradually increase, leading to the change of capsid conformation. When such a charge imbalance reaches over a certain threshold, the degree of change of capsid structure would allow the (transient) exposure of the HBc NLS to capsid exterior. The exposed NLS can then be recognized by cellular importin, and nuclear transport of HBc protein or particles can then be facilitated. According to this hypothesis, the NLS present in chimeric HBc reporters (Fig. 3–6) should be the same one present in HBc capsid particles with ongoing HBV DNA replication (Fig. 2).

Previously, we reported that HBc protein in the nucleus can exist as capsid particles [35], as it can be stained by using a monoclonal antibody Hyb-3120 specific for HBc capsid conformation [34]. Recently, it has been reported that nuclear entry of HBc capsids could involve disintegration of capsids to protein dimers, followed by nuclear reassociation of dimers to capsids [52]. If this scenario is true, the NLS associated with HBc capsids is likely to be the same one present in HBc protein monomers or dimers.

### Multiple layers of regulation of HBc subcellular localization

The nuclear targeting behavior of HBc mutant ARD-II+IV in Huh7 cells is consistent with that in the hydrodynamic mouse model. However, what could be an explanation for the higher frequency of nuclear localization of wild type HBc in the hydrodynamic mouse model (26% N>C pattern, Table 1, vs. 1% N>C pattern, Fig. 2)? We are not sure whether liver injury caused by the hydrodynamic injection could contribute in part to this phenomenon. When a control vector without HBV DNA was used for hydrodynamic injection (data not shown), vacuolating hepatocytes were often observed near the central vein (like the vacuolating hepatocytes in Fig. 8). Therefore, hepatic vacuolation is probably in part due to the physical shearing stress during the hydrodynamic injection. A similar phenomenon of vacuolating hepatocytes was observed previously after hydrodynamic delivery [53], [54]. It is also possible that a minor degree of hepatic injury could cause liver regeneration and mitosis of hepatocytes. Previously, it has been shown that cell cycling could influence nuclear localization of HBc in tissue culture [55]. In addition to the intrinsic factors, such as NLS and NES of HBc, extrinsic factors, such as inflammation, innate immunity, liver regeneration, cell cycling, and host factors (such as importin, TAP, cellular kinase), could all influence the ultimate localization of HBc. These extrinsic factors could be quite variable in activities in different assay systems (e.g., cell culture vs. animal models). Taken together, these extrinsic and intrinsic factors constitute multiple layers of regulation of intracellular HBc trafficking. As such, the distribution of HBc between nucleus and cytoplasm of various mutants is not simply categorical, but only relative. In this regard, it is particularly helpful to practice the double blind protocol in IFA and perform Western blot analysis.

### Biological significance of nucleocytoplasmic shuttling of HBc

**Figure 10:**
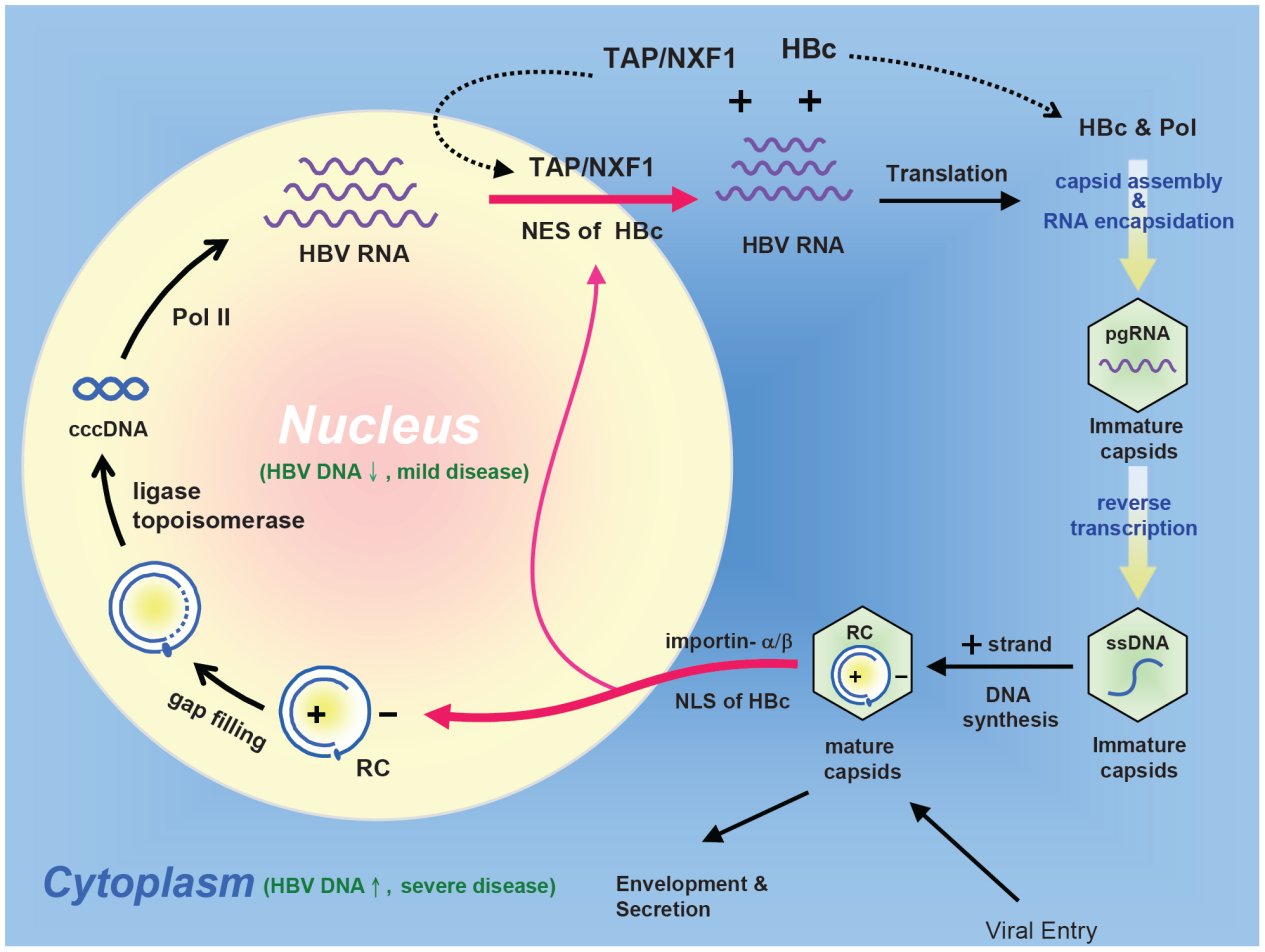
An intracellular loop of HBV ccc DNA amplification relies on the nucleocytoplasmic shuttling of HBc. RC: relaxed circle DNA, ccc: covalently closed circle DNA, pol: HBV-encoded polymerase, pol-II: cellular DNA-dependent RNA polymerase II, The nuclear import of HBV RC DNA, and the nuclear export of HBV pgRNA (pregenomic RNA) could all be facilitated by its dynamically shuttling HBc. Furthermore, an HBc trafficking hypothesis of acute liver exacerbation assumes that HBV DNA replication and disease activity are significantly suppressed when HBc is predominantly localized in the nucleus. In contrast, when HBc is predominantly localized in the cytoplasm, HBV DNA replication is activated, and liver disease activity is elevated.

TAP is known as a primary receptor for general mRNA export [28]. Recently, it has been demonstrated that TAP and ICP27 interaction as well as RNA binding by ICP27 are required for efficient nuclear export of herpes simplex virus 1 transcripts [27]. Similarly, Epstein-Bar virus encodes an early protein EB2 which contains an NES. This NES contains several small arginine rich domains which are important for TAP binding and EB2 shuttling [45]. Both EB2 and ICP27 behaved like a RNA binding adapter which could facilitate mRNA export by binding to TAP. The functional significance of nuclear HBc has been a mystery. As shown in Fig. 10, we speculate that HBc ARD could serve as a RNA binding adapter for nuclear export of HBV specific transcripts, based on the following rationales: 1) TAP is a nucleocytoplasmic shuttling protein [28]. 2) TAP is a known RNA and protein export receptor [27], [28], [45]. 3) TAP and HBc can bind to each other (Fig. 7A). 4) HBc nuclear export depends on TAP (Fig. 7B). 5) HBc can bind RNA through the ARD [56]. 6) HBc also appears to be a nucleocytoplasmic shuttling protein (Fig. 5 and 7B).

As outlined in Fig. 10, mature HBc capsids in the cytoplasm can import RC DNA into the nucleus, probably by complex association with the importin machinary. The imported RC DNA can be converted into covalently closed circular (ccc) DNA, which in turn serves as a template for pol-II mediated transcription. HBV pregenomic RNA (pgRNA) in the nucleus can then be exported to the cytoplasm by TAP and HBc complex. Translation of cytoplasmic HBV mRNA results in HBc and polymerase protein. RNA encapsidation of immature capsids occurs in the cytoplasm by complex association among polymerase, HBc, and pgRNA. Immature capsids gradually grow mature after reverse transcription [7]. Mature capsids can then repeat the cycle, and build up the ccc DNA pool in the nucleus [8]. The nucleocytoplasmic shuttling of HBc is thus central to the intracellular loop of ccc DNA amplification during the life cycle of HBV.

### Correlation between HBV replication and HBc subcellular localization

In Fig. 2D, both mutant ARD-II+IV and mutant ARD-I+III can replicate equally well by Southern blot analysis (Fig. 2D), yet their respective subcellular localizations are opposite to each other (Fig. 2A and 2B). Therefore, there is no apparent correlation between HBc subcellular localization and HBV DNA synthesis. In contrast, in Fig. 7F, knockdown of TAP resulted in HBc nuclear localization which is correlated with reduced HBV DNA and HBsAg synthesis. For the lack of a perfect correlation between nuclear localization of HBc and reduced viral DNA synthesis, our favorite explanation is that such a correlation exists only for the wild type-like HBV or its naturally occurring variants, but not true for artificially engineered arginine deficient mutants, such as mutant ARD-I+III or ARD-II+IV. As shown in Fig. 2D, the replication patterns between WT-HBV and ARD mutants are very different. The latter has a “short-DNA phenotype” [9], [11], which is absent in wild type HBV. Therefore, while the general correlation between nuclear localization of HBc and reduced viral DNA synthesis could hold true for wild-type like variants, it cannot be applied to engineered ARD mutants with significant charge imbalance [9], [10], [11]. In fact, very few naturally occurring HBc variants contain mutations at the arginine residues of HBc ARD [57]. The most frequent naturally occurring mutations of HBc occurred at the capsid assembly domains of HBc 1–149, particularly at hotspots of amino acids 5, 13, 86, 97, and 130.

### Potential clinical significance of HBc nucleocytoplasmic shuttling

To date, the molecular mechanism of acute exacerbation of chronic hepatitis B has remained unclear [58]. Similarly, the molecular mechanism of spontaneous relapse of hepatitis in asymptomatic HBsAg carriers has remained to be elucidated [59]. Here, we hypothesize that the unleash of HBc from nucleus to cytoplasm can be triggered by some unknown extrinsic factors, leading to the reactivation of HBV replication in the cytoplasm of hepatocytes and thus a new wave of immune attack and cytolysis from host defense (Fig. 10). In this context, we envision the nucleus as a reservoir for HBV genome and an “immune privileged site” for the shuttling HBV core protein.

### Accession numbers/ID numbers for genes and proteins mentioned in the text

The accession numbers in GenBank for hepatitis B virus (*ayw*) is V01460, in Entrez Gene for TAP/NXF1 is 10482, for SV40 LT is 1489531, for HIV-1 Rev is 155908, and in UniProtKB/Swiss-Prot for TAP/NXF1 is Q9UBU9, for SV40 LT is P03070, for HIV-1 Rev is P69718.

## Supporting Information

Figure S1 HBc ARD can function like a CRS (cytoplasmic retention signal). (**A**) Heterokaryon analysis suggests efficient nuclear import of wild type Rev protein (green) from transfected human cells (arrowhead) to untransfected mouse nuclei (arrow). Mouse nuclei can be differentiated from human nuclei by their brighter DAPI blue staining pattern. (**B**) Heterokaryon analysis suggests the lack of efficient nuclear import of Rev-HBc ARD chimera protein (green) from transfected human cells (arrowhead) to untransfected mouse nuclei (arrow). Mouse nuclei can be differentiated from human nuclei by their brighter DAPI blue staining pattern. HBc ARD can function like a CRS.
(5.26 MB TIF)

Figure S2 HBc ARD can function like an NES (nuclear export signal). SV40 LT is a nuclear protein which contains an NLS, but without any NES. Heterokaryon analysis suggests efficient transport of SV40LT-HBc ARD chimera protein from transfected human cells to untransfected mouse nuclei. arrowhead: human Huh7 cells transfected with SV40LT-HBc ARD chimera; arrow: unstransfected mouse NIH3T3 cells with a much brighter DAPI blue staining pattern characteristic of mouse origin.
(2.55 MB TIF)

Figure S3 HBc ARD can function as a CRS or NES, depending on the relative strength between NLS and NES in the same context. (**A**) SV40 LT is a nuclear protein which contains an NLS, but without any NES. Upon fusion with HBc ARD, the chimera of SV40LT-HBc can be transported from transfected human to untransfected mouse nuclei by heterokaryon analysis. Arrowhead: human Huh7 cells cotransfected with SV40LT-HBc ARD chimera (red) and wild type Rev (green); arrow: unstransfected mouse NIH3T3 cells with mouse characteristic brighter DAPI blue staining. (**B**) Heterokaryon analysis suggests that SV40LT-HBc chimera can be transported from transfected human to untransfected mouse nuclei. This result suggests the existence of an NES in HBc ARD. In contrast, Rev-HBc is localized only to the cytoplasm, suggesting the existence of a CRS in HBc ARD. We speculate that the same HBc ARD can function as a CRS or NES depending on the relative strength between NLS and NES in the same context. Arrowhead: human Huh7 cells cotransfected with SV40LT-HBc chimera (red) and Rev-HBc chimera (green). Arrow: unstransfected mouse NIH3T3 cells with mouse characteristic brighter DAPI blue staining.
(4.73 MB TIF)

Figure S4 The NES of HBc ARD is associated with ARD-II and ARD-IV. Heterokaryon analysis: Huh7 cells transfected with SV40LT-HBc ARD-II+IV chimera were fused with NIH-3T3 cells. Lack of transport from human to mouse nuclei was noted. This result suggests that the NES of HBc resides in ARD-II and ARD-IV. Arrowhead: human Huh7 cells transfected with SV40LT-HBc ARD-II+IV (green). Arrow: unstransfected mouse NIH3T3 cells with mouse characteristic brighter DAPI blue staining.
(2.26 MB TIF)

Figure S5 The subcellular distribution of the Rev protein is insensitive to the treatment with si-TAP. Treatment with siRNA specific for TAP has no significant effect on the subcellular localization of wild type Rev protein in Huh7 cells transfected with plasmid pCMV-Rev (green). This result served as a control to Fig. 7B, and it argues for a specific effect of si-TAP treatment on the nuclear accumulation of HBc protein in Huh7 cells transfected with a wild type HBV replicon pCH93091 in Fig. 7B.
(0.60 MB TIF)
