# Supplementary figures and images for: Nuclear Export and Import of Human Hepatitis B Virus Capsid Protein and Particles
Source: PLoS Pathog. 2010 Oct 28;6(10):e1001162. doi: 10.1371/journal.ppat.1001162 (PMC2965763; doi:10.1371/journal.ppat.1001162)

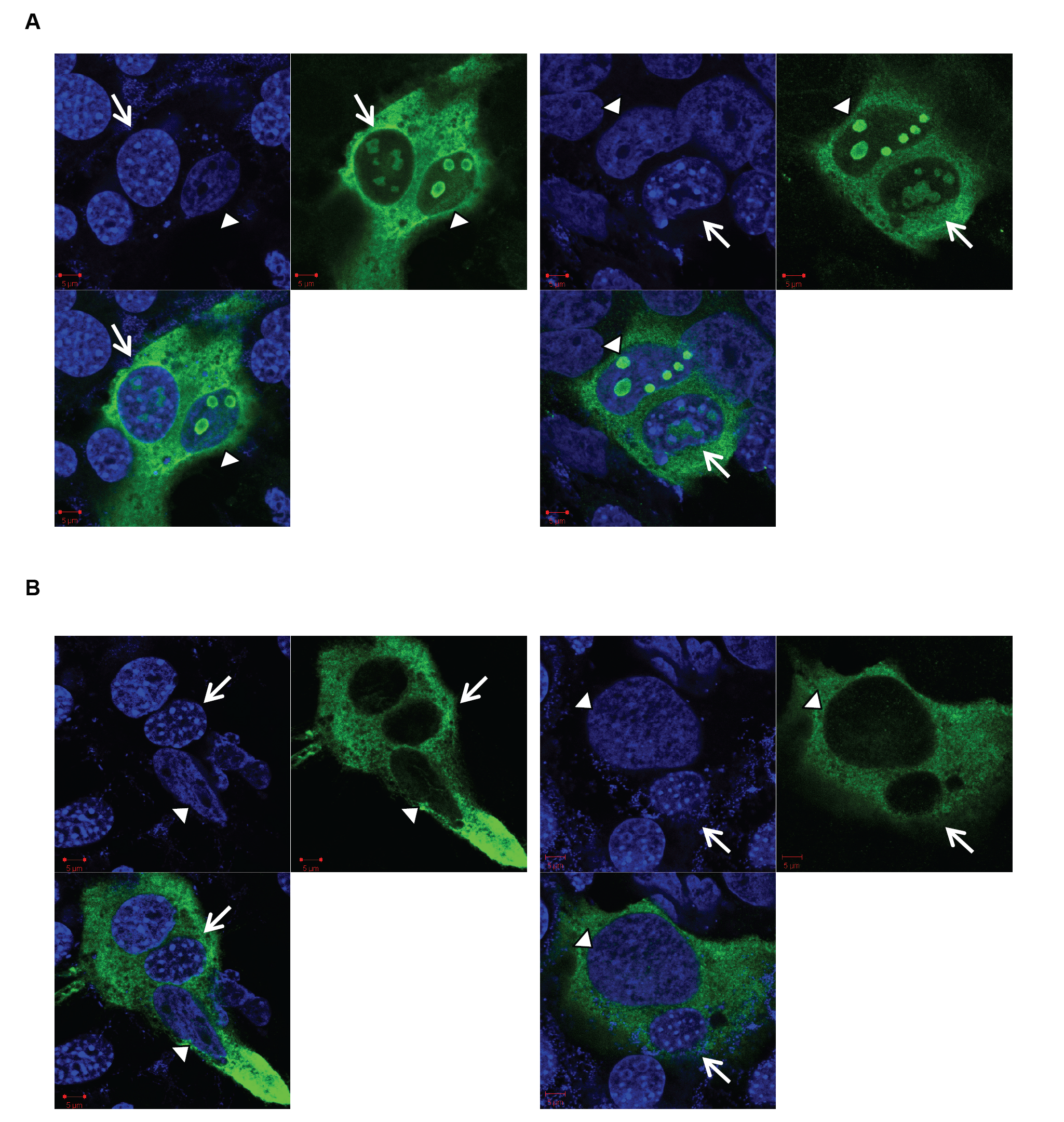

Supplement: Figure S1 — HBc ARD can function like a CRS (cytoplasmic retention signal). (A) Heterokaryon analysis suggests efficient nuclear import of wild type Rev protein (green) from transfected human cells (arrowhead) to untransfected mouse nuclei (arrow). Mouse nuclei can be differentiated from human nuclei by their brighter DAPI blue staining pattern. (B) Heterokaryon analysis suggests the lack of efficient nuclear import of Rev-HBc ARD chimera protein (green) from transfected human cells (arrowhead) to untransfected mouse nuclei (arrow). Mouse nuclei can be differentiated from human nuclei by their brighter DAPI blue staining pattern. HBc ARD can function like a CRS. (5.26 MB TIF) [file ppat.1001162.s001.tif]

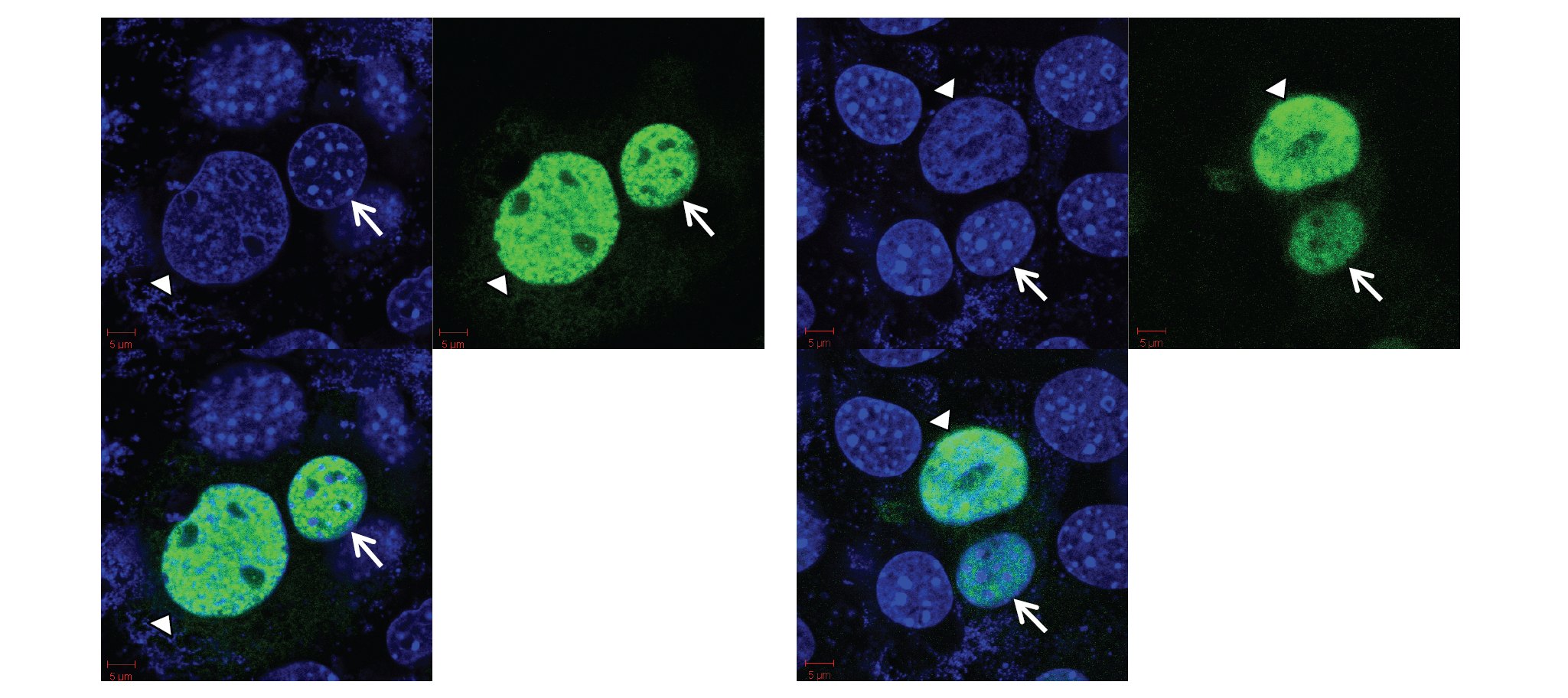

Supplement: Figure S2 — HBc ARD can function like an NES (nuclear export signal). SV40 LT is a nuclear protein which contains an NLS, but without any NES. Heterokaryon analysis suggests efficient transport of SV40LT-HBc ARD chimera protein from transfected human cells to untransfected mouse nuclei. arrowhead: human Huh7 cells transfected with SV40LT-HBc ARD chimera; arrow: unstransfected mouse NIH3T3 cells with a much brighter DAPI blue staining pattern characteristic of mouse origin. (2.55 MB TIF) [file ppat.1001162.s002.tif]

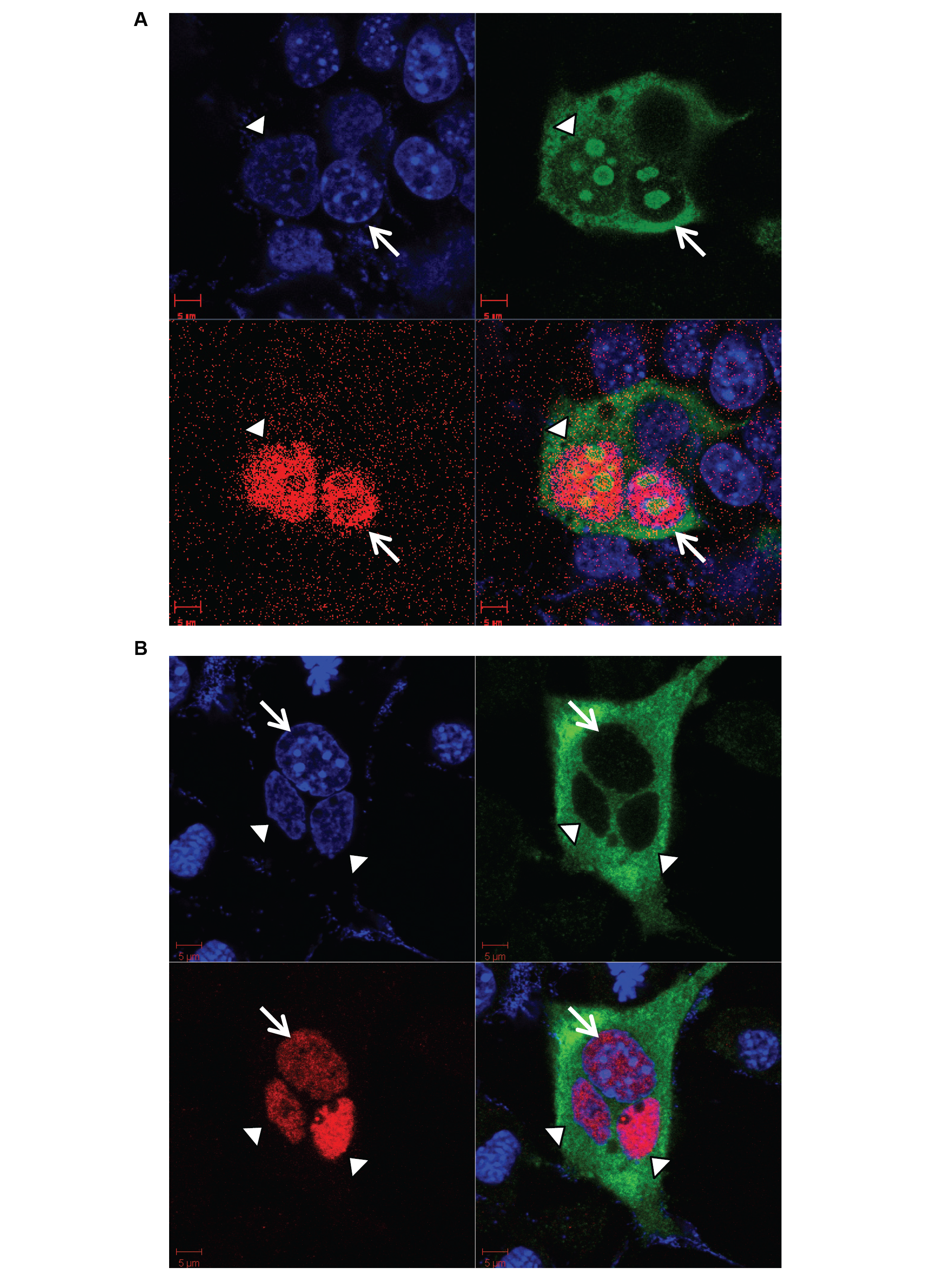

Supplement: Figure S3 — HBc ARD can function as a CRS or NES, depending on the relative strength between NLS and NES in the same context. (A) SV40 LT is a nuclear protein which contains an NLS, but without any NES. Upon fusion with HBc ARD, the chimera of SV40LT-HBc can be transported from transfected human to untransfected mouse nuclei by heterokaryon analysis. Arrowhead: human Huh7 cells cotransfected with SV40LT-HBc ARD chimera (red) and wild type Rev (green); arrow: unstransfected mouse NIH3T3 cells with mouse characteristic brighter DAPI blue staining. (B) Heterokaryon analysis suggests that SV40LT-HBc chimera can be transported from transfected human to untransfected mouse nuclei. This result suggests the existence of an NES in HBc ARD. In contrast, Rev-HBc is localized only to the cytoplasm, suggesting the existence of a CRS in HBc ARD. We speculate that the same HBc ARD can function as a CRS or NES depending on the relative strength between NLS and NES in the same context. Arrowhead: human Huh7 cells cotransfected with SV40LT-HBc chimera (red) and Rev-HBc chimera (green). Arrow: unstransfected mouse NIH3T3 cells with mouse characteristic brighter DAPI blue staining. (4.73 MB TIF) [file ppat.1001162.s003.tif]

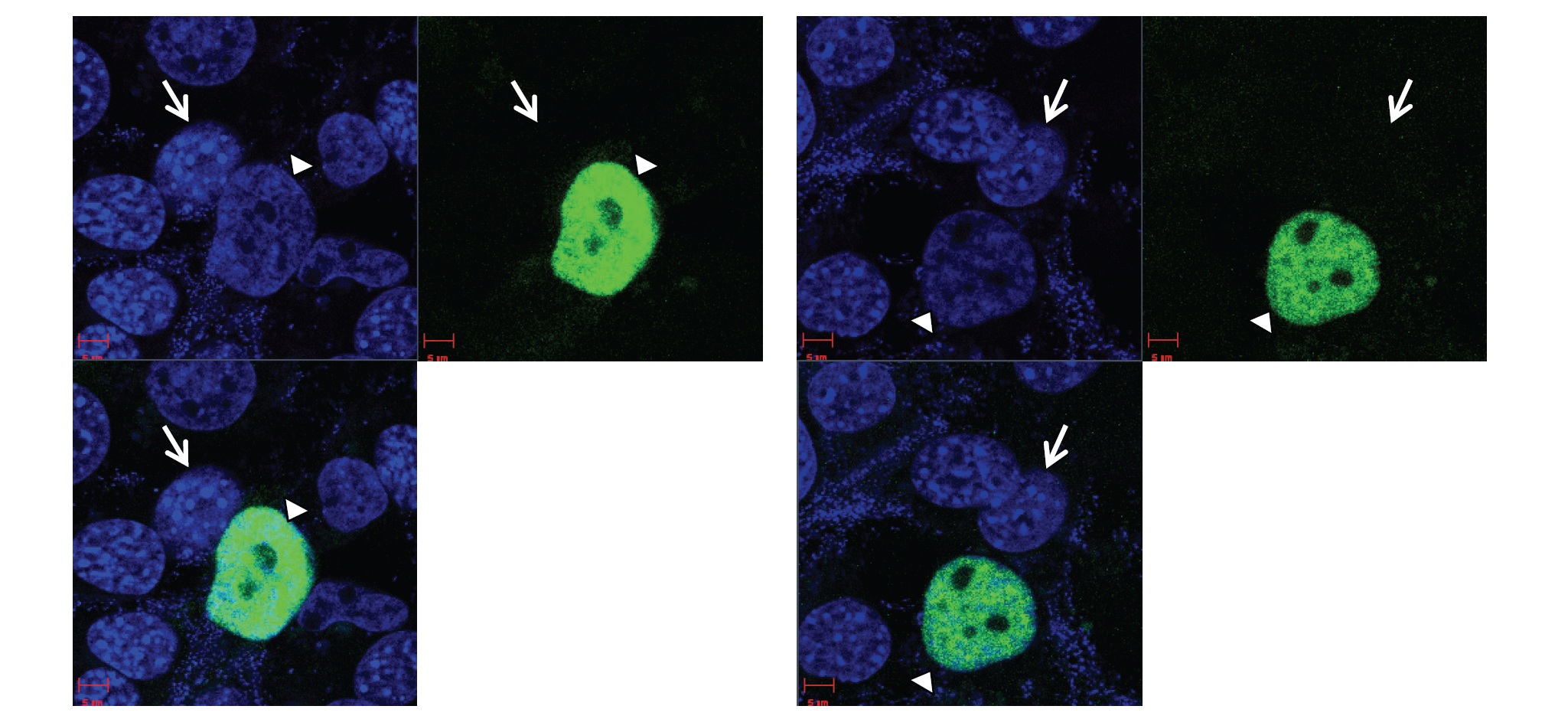

Supplement: Figure S4 — The NES of HBc ARD is associated with ARD-II and ARD-IV. Heterokaryon analysis: Huh7 cells transfected with SV40LT-HBc ARD-II+IV chimera were fused with NIH-3T3 cells. Lack of transport from human to mouse nuclei was noted. This result suggests that the NES of HBc resides in ARD-II and ARD-IV. Arrowhead: human Huh7 cells transfected with SV40LT-HBc ARD-II+IV (green). Arrow: unstransfected mouse NIH3T3 cells with mouse characteristic brighter DAPI blue staining. (2.26 MB TIF) [file ppat.1001162.s004.tif]

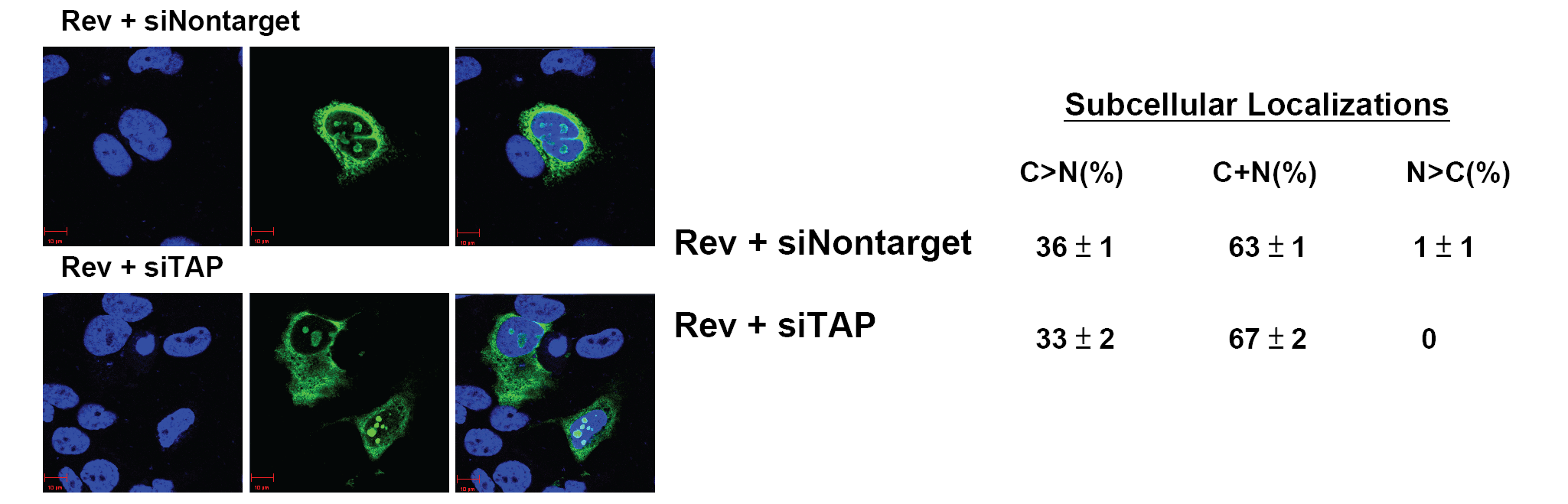

Supplement: Figure S5 — The subcellular distribution of the Rev protein is insensitive to the treatment with si-TAP. Treatment with siRNA specific for TAP has no significant effect on the subcellular localization of wild type Rev protein in Huh7 cells transfected with plasmid pCMV-Rev (green). This result served as a control to Fig. 7B, and it argues for a specific effect of si-TAP treatment on the nuclear accumulation of HBc protein in Huh7 cells transfected with a wild type HBV replicon pCH93091 in Fig. 7B. (0.60 MB TIF) [file ppat.1001162.s005.tif]
